# Safety of Plastic Food Packaging: The Challenges about Non-Intentionally Added Substances (NIAS) Discovery, Identification and Risk Assessment

**DOI:** 10.3390/polym13132077

**Published:** 2021-06-24

**Authors:** Lilian Seiko Kato, Carlos A. Conte-Junior

**Affiliations:** 1 Center for Food Analysis (NAL), Technological Development Support Laboratory (LADETEC), Federal University of Rio de Janeiro (UFRJ), Cidade Universitária, Rio de Janeiro 21941-598, Brazil; conte@iq.ufrj.br; 2 Laboratory of Advanced Analysis in Biochemistry and Molecular Biology, (LAABBM), Department of Biochemistry, Federal University of Rio de Janeiro (UFRJ), Cidade Universitária, Rio de Janeiro 21941-909, Brazil; 3 Graduate Program in Food Science (PPGCAL), Institute of Chemistry (IQ), Federal University of Rio de Janeiro (UFRJ), Cidade Universitária, Rio de Janeiro 21941-909, Brazil; 4 Graduate Program in Veterinary Hygiene (PPGHV), Faculty of Veterinary Medicine, Fluminense Federal University (UFF), Vital Brazil Filho, Niterói 24220-000, Brazil; 5 Graduate Program in Sanitary Surveillance (PPGVS), National Institute of Health Quality Control (INCQS), Oswaldo Cruz Foundation (FIOCRUZ), Rio de Janeiro 21040-900, Brazil; 6 Graduate Program in Chemistry (PGQu), Institute of Chemistry (IQ), Federal University of Rio de Janeiro (UFRJ), Cidade Universitária, Rio de Janeiro 21941-909, Brazil

**Keywords:** food packaging additives, food contact materials (FCMs), food safety, additives in polymers, migration study, food contact articles (FCAs)

## Abstract

Several food contact materials (FCMs) contain non-intentionally added substances (NIAS), and most of the substances that migrate from plastic food packaging are unknown. This review aimed to situate the main challenges involving unknown NIAS in plastic food packaging in terms of identification, migration tests, prediction, sample preparation, determination methods and risk assessment trials. Most studies have identified NIAS in plastic materials as polyurethane adhesives (PU), polyethylene terephthalate (PET), polyester coatings, polypropylene materials (PP), multilayers materials, plastic films, polyvinyl chloride (PVC), recycled materials, high-density polyethylene (HDPE) and low-density polyethylene (LDPE). Degradation products are almost the primary source of NIAS in plastic FCMs, most from antioxidants as Irganox 1010 and Irgafos 168, following by oligomers and side reaction products. The NIAS assessment in plastics FCMs is usually made by migration tests under worst-case conditions using food simulants. For predicted NIAS, targeted analytical methods are applied using GC-MS based methods for volatile NIAS and GC-MS and LC-MS based methods for semi- and non-volatile NIAS; non-targeted methods to analyze unknown NIAS in plastic FCMs are applied using GC and LC techniques combined with QTOF mass spectrometry (HRMS). In terms of NIAS risk assessment and prioritization, the threshold of toxicological concern (TTC) concept is the most applied tool for risk assessment. Bioassays with sensitive analytical techniques seem to be an efficient method to identify NIAS and their hazard to human exposure; the combination of genotoxicity testing with analytical chemistry could allow the Cramer class III TTC application to prioritize unknown NIAS. The scientific justification for implementing a molecular weight-based cut-off (<1000 Da) in the risk assessment of FCMs should be reevaluated. Although official guides and opinions are being issued on the subject, the whole chain’s alignment is needed, and more specific legislation on the steps to follow to get along with NIAS.

## 1. Introduction

Food packaging can contain non-intentionally added substances (NIAS) as a result of the interactions between different substances in the packaging materials, between food content and substances (for example, additives) in food contact material (FCM), from degradation processes and mainly from the impurities present in the raw materials used for FCM production [1,2,3,4,5]. (EU) nº 10/2011 defines that “non-intentionally added substance means an impurity in the substances used or a reaction intermediate formed during the production process or a decomposition or reaction product”. Most NIAS are regularly detected when using high sensitivity analytical techniques, although the chemical structure of unknown compounds is often difficult to establish by conventional tools [1,4,5]. Due to the increasing complexity of FCMs and food contact articles (FCAs), NIAS will stay an essential topic in the coming years [6]. Their detection and identification are becoming easier due to advances in analytical techniques and growing databases [5].

Regulatory authorities worldwide recognized the importance of a proper risk assessment for NIAS. The development of practical guidelines that guarantee the safety of FCMs, including their NIAS, is intensively discussed by industry, the scientific community and regulatory agencies [7,8]. The EU legislation is a positive list-based system, and only substances authorized and listed in the specific measures may be used. The European Commission legalizes substances for the application in FCMs, and there are substances exempted from positive listing including colorants, polymer production aids (PPA’s), solvents, aids to polymerization (AP’s), oligomers and the so-called NIAS, that could be present in the FCMs and may migrating to the food. NIAS includes oligomers, impurities and contaminants from the raw materials or the production processes, reaction by-products from authorized substances (additives) and manufacturing of packaging materials, decomposition or degradation products from authorized substances [9,10]. The challenge was created: what to do in the inevitable event of a material having detectable NIAS amounts?

According to the Scientific Opinion published on January 29, 2016, by the EFSA [8], The Union list of authorized substances in Regulation (EU) nº 10/2011 does not include what has been called the NIAS: oligomers, reaction products and impurities. The regulation declares that NIAS should be considered in the risk assessment of plastic FCM and included, if necessary, in a substance’s specifications. As the NIAS often constitutes the central part of the migration, a more detailed reflection of these is necessary, including considering the authorized substances’ manufacturing and use conditions and plastics. Substances that migrate into foodstuffs require equal treatment in risk assessment, irrespective of their source or intended function. For all those not explicitly regulated substances, if they migrate to food and if used intentionally or are NIAS, the package manufacturers and food industries must demonstrate safety in their supporting documentation [8].

In the US, according to 21 CFR 170.3 [11], any food contact substance (FCS) expected to migrate into food through the FCA must conform with the legal requirements. An FCS is “any substance that is intended for use as a component of materials used in manufacturing, packing, packaging, transporting, or holding food if such use is not intended to have any technical effect in such food”. This definition does not cover non-intentionally added substances, and the term NIAS is not applied in a legal context in the US. However, there are several provisions concerning some NIAS types, e.g., the safety assessment shall also include “any substance formed in or on food because of its use”. Under 21 CFR 174.5 [11], any FCS “shall be of a purity suitable for its intended use” and, also in the case of polymers, the submission of information on the significant impurities and side reactions is recommended. The US Food and Drug Administration agency (FDA) is facing increasing pressure about the presence of impurities in food contact materials, with congressional representatives requesting to initiate a review of FDA procedures [12].

In this context, despite all these needs and requirements, the paths to follow are challenging, and the challenges are continuous in dealing with impurities/contaminants in raw materials and polymer processing machines, side products originated from the synthesis of additives and manufacturing of packaging materials and degradation products of additives and packaging materials [10]. This review aims to situate the main challenges in identifying and determining NIAS in plastic food packaging, seeking to direct the following steps to achieve the agencies’ official regulation and application and adequacy of the food industries.

## 2. Plastic food Packaging and Legislation

Nowadays, it is impossible to separate food from packaging since packaging offered protection from physical damage, soiling and microbial spoilage and has now been a challenge for the industry and, consequently, studies focus on its safety. Over the last decades, food contact materials (FCMs) and food contact articles (FCAs) use have increased exceptionally, with a trend towards smaller packs with a larger contact surface, more processed foods with long-term storage and products heated in the packaging. The legislation’s regulators often become outdated because risk assessment (RA) studies take time and are difficult to perform.

Polymers are one of the essential FCMs. Plastics packaging materials (PPMs) are emerging as the primary material used in food packaging due to their functional properties, convenience and low costs [13,14,15]. Additives are introduced into polymers to improve their physical and chemical properties [16,17], such as increased resin stability to oxidation and light exposure, impact resistance, hardness increase or decrease, surface tension control, reduced costs and increased flame resistance [18]. The significant families of polymer additives are fillers, plasticizers, flame-retardants, colorants, lubricants, foaming agents, antistatic agents and stabilizers divided into groups with more specific antioxidants antiozonants, heat stabilizers, UV stabilizers and biocides [19]. Consequently, all additives must be certified by a specific regulatory authority since FCMs are a significant source of chronic exposure to chemicals [20].

The establishment of strict regulations regarding the safety and quality of FCMs and FCAs by countries is primordial. In the European Union (EU), the European Food Safety Authority (EFSA) promotes scientific advice to the legislator (Commission) for authorizing substances used for plastics. The European Commission (EC), in turn, establishes rules and regulations for food contact packaging. The EC’s essential specific measure is the Regulation (EU) nº 10/2011 [21] on plastic materials in contact with food containing a positive listing of additives, monomers, starting substances, macromolecules obtained from microbial fermentation additives. The regulation received a recent amendment on 23 September 2020. In the United States (US), food contact packaging safety is set by the Food and Drug Administration (FDA), through Code of Federal Regulations Title 21, Chapter I, Subchapter B, 177.1010 to 177.2910 on Indirect Food Additives-Polymers and sections 174.5 on General provisions applicable to indirect food additives and 174.6 on Threshold of regulation for substances used in food-contact articles [11]. 

In July 2019, the Common Market of the South (Mercosur), a trade bloc that represents multiple countries in Latin America, published a resolution (GMC 39/19) listing additives allowed for plastics and polymeric coatings for use in FCMs [22]. On 3 December 2019, the National Health Surveillance Agency (ANVISA) from Brazil, officially published the resolution RDC No. 326, establishing the positive list of additives for the preparation of plastics and polymeric coatings in contact with food and other measures. This resolution incorporates the (GMC)/MERCOSUR n° 39/19 to the national legal system. Food contact plastics are also governed by GMC Resolution Numbers 02/12 (positive list of monomers and polymers), 56/92 (general provisions and overall migration limits) and 32/10 (framework test conditions). 

On 9 August 2019, Japan notified the World Trade Organization (WTO) of its draft Positive List (PL) System for food-contact plastics (WTO Notification G/TBT/N/JPN/630). In China, the China Food Safety Law regulates FCMs since 2009. The most crucial Chinese food safety standard for food contact additives is GB 9685-2016 on National Food Safety Standard: Standard for Use of Additives in Food Contact Materials and Articles. National Health Commission of the People’s Republic of China—NHC has issued 13 announcements until August 2019, including the approvals of 90 additives used for FCMs and articles (38 of them are food contact additives with an expanded scope or using amount).

The consequences of the interactions between food and packaging are diverse. FCM is an underestimated chemical contaminant source and a potentially relevant route of human exposure to organic and inorganic substances. Thus, evaluating migrants in food are a continual study. Contamination of food by the migration of plastic additives and their degradation products is a relevant matter for health legislation due to potential health-related risks. After assessing the consumer’s absence of toxic effects, packaging materials intended for foodstuffs packaging must be approved (EU Regulation 1935/2004). This is still a subject of debate due to the lack of information and more detailed studies on the safety of exposure to these possible contaminants [23]. 

## 3. Migration of Substances from Packaging to the Food

The FCMs, depending on the circumstances, may transfer their constituents to the foodstuffs. This mass transfer phenomenon is called migration, leading to high human exposure to certain chemicals [24,25,26,27]. Considerable knowledge concerning the migration potential of FCMs has been accumulated recently, primarily in support of the FDA, European Commission and international food contact materials legislation and the scientific community [28,29,30,31,32,33,34]. The migration can occur from packaging to the food, where molecularly diffused substances of low molecular weight (e.g., oligomers or additives) can be transferred into foods [35] and from food to the packaging [36].

**Figure 1:**
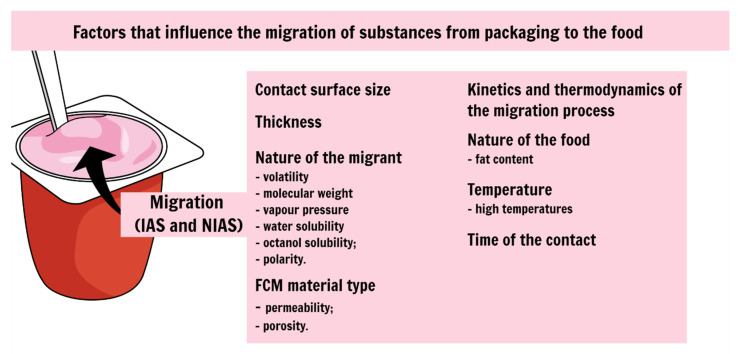
Main factors that influence the migration of substances from packaging to food.

Migration is of significance for smaller size compounds (below 1000 Da) [37,38]; despite in terms of risk assessment, oligomers up to a molar weight of 1000 Da are evaluated to be relevant under the assumption of human gastrointestinal absorption [39,40]. The dimension to which migration occurs depends on several factors (Figure 1) listed below [41,42,43]:

- the contact surface size, since the more extensive the contact surface between packaging and food, the higher the migration rate [24];
- the nature of the migrant, since the more volatile and the lower the molecular weight, the greater the migration rate (also the vapor pressure, water solubility, octanol solubility and polarity) [44];
- FCM material type (e.g., impermeable, permeable, porous materials) [45];
- kinetics and thermodynamics of the migration process (how fast will a substance transfer from the FCM into food) [46,47];
- the nature of the food, since high-fat foods are reported with a high level of migration, for example [42,45];
- temperature, high temperatures demonstrate high migration rates [42,44];
- contact during serving and duration of the time of contact [42,45].

In the specific case of plastics, non-inert materials, the migration can occur from the inner side of the packaging and internal layers due to diffusion processes [18,48,49]. According to the deterministic model, the migration of substances from packaging to food occurs through diffusion, where there is a movement of molecular structures from the high concentration to the low concentration region until equilibrium is reached. Fick’s second law can describe the diffusion rate [10,50,51,52,53]. Some studies describe precisely how to use this mathematical model [42,47,53]. In addition to diffusion, there is also desorption of diffuse polymer surface molecules, sorption of compounds at the plastic-food interface and desorption of compounds in the food that describe the migration stages [24]. 

Migration modelling can complement or substitute actual laboratory migration experiments that are expensive and time-consuming. Thus, it has become a popular tool for researchers, industry and regulators to predict migration. Efforts have been made to modify the standard “worst-case” deterministic models to reflect more realistic migration scenarios better. These approaches comprehend probabilistic and stochastic modelling that considers variability and uncertainty in the mass transfer parameters, mechanistic or empirical migration models that can be combined with food consumption data to estimate dietary exposure to the chemicals [17,54,55,56]. However, trained specialists should only use migration models with in-depth knowledge of chemical migration to make reliable predictions and correctly interpret the results. Gavriil et al. [55] published an extensive review to evaluate the migration models’ efficiencies, concluding that these models cannot predict the total accurate migration from a different FCM to the food volume since the material might contain several completely unknown compounds. Thus, the migration studies are still the traditional method for evaluating the migration of substances from packaging to food, despite analytical challenges and cost.

Two terms of high significance that should not be confused are the overall migration (OM) and the specific migration (SM). The OM refers to the sum of all mobile substances of packaging released per unit area of the package under the influence of specific predetermined conditions. On the other hand, SM is only related to a specific known substance [57]. 

In most cases, and as provided by legislation ((EU) n° 10/2011), the migration study is performed with food simulants in place of the food itself, due to the high complexity that food has typically as a matrix than foods [29,58,59]. They should represent the significant physicochemical properties exhibited by each food type. When using food simulants, temperature and standard testing time should replicate the migration from the FCM into the food [42,60]. Moreover, current legislation provides for the simulation of temperature and contact period situations in which migration tests performed according to the actual situation’s food storage conditions. 

In the US, according to The Guidance for Industry: Preparation of Premarket Submissions for Food Contact Substances (Chemistry Recommendations) published by FDA in 2007, sponsors should provide information sufficient to permit estimation of the daily dietary consumption of the food contact substances (FCS), e.g., consumer exposure. The FDA will calculate the FCS consumption or other components consumption that might migrate to food expected daily based on analyzed or estimated levels in food or food simulants. Still following the guidance, “Although the FDA always has accepted reliable analyses of FCS in real foods, many analytes are difficult to measure in food in practice. As an alternative, sponsors may submit migration data acquired with food simulants to reproduce the FCS’s nature and migration into food. Because an FCS can contact many foods with different processing conditions and shelf lives, the submitted migration data should reflect the most severe temperature/time conditions to which the food contact article (FCA) containing the FCS will be exposed. The document brings a table with the respective recommended simulant according to the type of food. The recommendation for aqueous and acidic foods is 10% ethanol; low- and high-alcoholic foods, 10–50% ethanol; fatty food, food oil (e.g., corn oil), HB307, Miglyol 812 or others. 

Two steps are involved in migration tests of substances from food packaging into food simulants: to expose the polymer packaging to the food simulant; to quantify the migrants transferred to a food simulant in terms of OM or SM. Determining OM is a regulatory requirement in EU countries with established migration limits for FCMs substances [24]. 

Most of the substances that migrate from packaging to food are unknown. A recent Scientific Opinion issued by the European Food Standard Authorization (EFSA) in 2016 comments that regarding identifying and evaluating all migrating substances, experience has shown that it is necessary to focus more on finished materials and articles used during the manufacturing process. Substances used in the manufacture of FCM or objects may contain impurities originating in their manufacture. Besides, during manufacture and use, reaction and degradation products may be formed, of which oligomers may be of the dominant class. These substances have become known as NIAS and referred to as such in the Commission Regulations. Regardless of whether they are intentional or unintended, all migrant substances’ safety, not just initiating substances—e.g., monomers or additives only—needs to be assessed, and the guidelines should be updated to account for this broader approach fully. This change to the finished FCM and its use requires an adjustment of the current substance listing system. The present work will discuss the definition, different aspects and types of NIAS, analytical methods of determination and quantification and the next section’s risk assessment task. 

## 4. NIAS in Plastic Food Packaging

**Figure 2:**
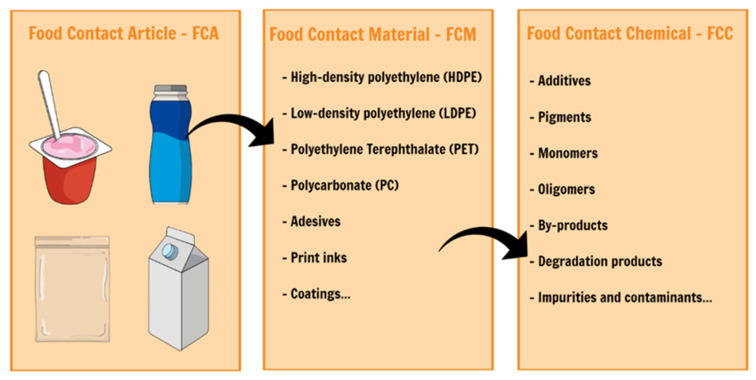
Illustration of key terms: FCA, FCM and FCC, and everything that makes up a food plastic packaging (adapted from Muncke et al. [23]).

Inventory lists of plastic FCMs contain several thousands of chemicals, including starting substances like monomers, production aids and additives [19,20,61]. However, these chemicals often suffer transformations, and final FCAs and FCMs may contain novel compounds that can migrate into food [19,61,62]. Figure 2 introduces the scientific terms for food contact articles (FCA), which are the combination of diverse food contact materials (FCMs) and food contact chemicals (FCCs) defined as substances used or present in the manufacture of FCMs or existing in the final FCMs or FCAs. Some FCCs are starting substances that no longer exist in the final FCM/FCA. Some FCCs are generated during the manufacture of an FCM/FCA or during the application of high temperatures, irradiation or the consumer’s food packaging. FCCs comprehend intentionally added substances (IAS), such as monomers, additives, catalysts and production aids; and impurities and reaction products (oligomers, polymers, by-products and degradation products), be referred to as non-intentionally added substances (NIAS) [7].

The FDA classifies Food Contact Substance (FCS) as “any substance that is intended for use as a component of materials used in manufacturing, packing, packaging, transporting or holding food if such use of the substance is not intended to have any technical effect in such food”. FCS could be “a single substance, such as a polymer or an antioxidant in a polymer. As a substance, it is reasonably pure”. Although a polymer may be composed of diverse monomers, it has a well-defined composition. FCM is “made with the FCS and (usually) other substances. It is often (but not necessarily) a mixture, such as an antioxidant in a polymer. The composition may be variable”.

It is impossible to know all substances that may be formed by the degradation of additives or plastics’ polymerization [1,19]. Their presence is often not known by the consumer or by the manufacturer [5,19]. If, according to Regulation EC 1935/2004 on materials and articles intended to come into contact with food, the manufacturer must ensure the safety of food contact packaging, it is clear that it is necessary to know about the NIAS formed and also to assess the risk assessment (RA) of these new substances. Still, following EC (EU) n° 10/2011, unauthorized substances may be used in FCMs plastics behind a functional barrier, providing they do not migrate to the food at levels above 10 µg/kg. Thus, in Europe, NIAS could be within this approach [5].

In the United States, according to the FDA Code Federal Regulation Title 21 Sec. 170.39 on the threshold of regulation for substances used in food-contact articles, substances that may enter the diet at levels below 50 µg/kg and demonstrate that they are non-genotoxic, are exempt from need authorization indirect food additive [11]. The FDA uses the term “Indirect Food Additive”, a food additive coming into contact with food as part of the packaging or processing but is not intended to be added directly to, become a constituent or have a technical outcome in/on the food. Indirect food additives cited in Title 21 of the US Code of Federal Regulations (21CFR) used in food-contact articles comprise adhesives and components of coatings (Part 175), paper and paperboard (Part 176), polymers (Part 177) and adjuvants and production aids (Part 178).

In this sense, the scientific community has been striving to define NIAS identification and determination strategies in FCMs and FCAs in general and strategies to predict the migration rate of these compounds from packaging to food and evaluate the health risks of each identified NIAS. A literature search was made using Medical Subject Headings (MeSH) terms on the Pubmed, Web of Science and Scopus databases. The initial screening process was performed from August to September 2020. Further directed searches were carried out by checking the reference lists of relevant articles. In this context, we defined a strategy composed of four phases to build the search strings: (i) identification of keywords considering the research question; (ii) synonyms based on relevant studies about non-intentionally added substances and plastic food packaging; and (iii) use of “AND” and “OR” Booleans operators.

**Figure 3:**
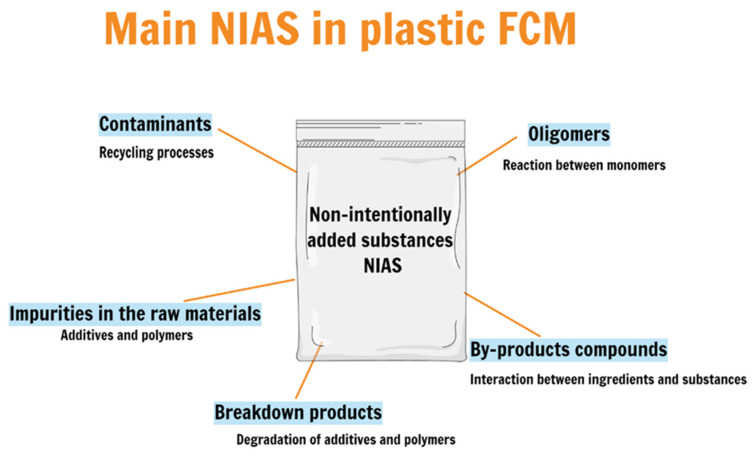
Mainly examples of NIAS formed during food plastic contact materials manufacturing, during the foodstuff’s shelf life, or during some processing (i.e., heating or irradiation).

- Search component 1 (SC1) was a population search: “food packaging” OR “food contact article” OR “food contact material” OR “packaging, food” OR “food containers” OR “plastic packaging materials” OR “multilayer food packaging” OR “multilayer packaging materials” OR “recycled plastic packaging” OR “acrylic adhesives” OR “recycled expanded polystyrene containers” OR “polyester-polyurethane” OR “food packaging polymer” OR “biodegradable food packaging” OR “nylons” OR “polyethylenes” OR “polypropylenes” OR “polystyrenes” OR “polyurethanes” OR “polyolefins” OR “acrylic adhesives” OR “recycled expanded polystyrene containers” OR “polyester-polyurethane” OR “polyvinyls” OR “polyesters” OR “polyethylene terephthalates” OR “polyhydroxy ethyl methacrylate” OR “silicones” OR “elastomers” OR “polyvinyl chloride” OR “silicone elastomers”.
- Search component 2 (SC2) was an intervention search: “non-intentionally added substances” OR “non-intentionally added compound” OR “NIAS” OR “breakdown products” OR “impurities” OR “side products” OR “neo-formed compounds” OR “degradation of polymers” OR “degradation of compounds” OR “degradation products” OR “non-volatile migrants” OR “volatile compounds” OR “non-volatile compounds” OR “volatile organic compound” OR “polymer additives” OR “oligomers” OR “additives” OR “plastic additives” OR “additive”.
- Because the ScienceDirect database only allows the use of a maximum of thirteen keywords in the search string, for this base it was used the following search components:
- SC1: (“food packaging” OR “food contact article” OR “food contact material” OR “plastic packaging materials”).
- SC2: (“non-intentionally added substances” OR “non-intentionally added compound” OR “NIAS” OR “additives”). Table 1 presents published works that identified NIAS on different food plastic packaging, the techniques used in each work and the migration tests applied. Despite all efforts, there is much to discover and to do in this area. Below are the leading examples of possible NIAS formation, shown in Figure 3.

### 4.1. Oligomers

Monomers’ reactions may cause oligomer formation during the polymerization processes due to incomplete polymerization, side reaction products like cyclic oligomers and subsequent thermal or hydrolytic degradation [105]. “Oligomers” are defined here as substances consisting of a small number (<20) of repeating units. Oligomers can also intentionally be used as “prepolymers”. These are reactive species that will be utilized as reacting blocks to manufacture polymers. When oligomers are not intentionally added, they are well within the scope of the NIAS definition, according to [7]. Oligomers do not come just from conventional polymers but also biodegradable polymers, and biopolymers also have a series of oligomers that can migrate to the food [5]. Despite this, according to other authors [105], the classification of oligomers as NIAS is still controversial due to similar properties of a homolog series of oligomers. Because the oligomer chemistry is linked to a polymer manufacturing process, it makes much sense to classify oligomers as polymer specific substances and not as NIAS. They are not explicitly regulated in the Regulation (EU) 10/2011. The extension of the EU positive list with new co-monomers as a consequence of industrial developments gives rise to an exponentially growing number of new oligomers present in food contact polymers as potential migrants.

However, according to a Scientific Opinion of EFSA (2016) [8], oligomers have become NIAS. Whether their presence is intended or not, it is needed to evaluate all migrating substances’ safety and not just the starting substances—e.g., the monomers or additives alone—and the guidelines should be updated. Importantly, oligomers are considered part of polymers in the US, with no impurities. Regulation (EU) No. 10/2011 does not explicitly regulate oligomers’ levels in general. However, two EFSA opinions concerning new co-monomers for polyester food-contact materials specified 50 µg kg^−1^ for total oligomer migration with less than 1000 Da [106,107]. The European Food Safety Authority (EFSA) states that the PA oligomers should not exceed 5 ppm (mg kg^−1^ food). Table 2 listed scientific publications that determined oligomers in different types of plastic food packaging. Most of the FCMs studied are polyester coatings and resins [29,63,64,108] and polyethylene terephthalate materials [28,65,66].

### 4.2. By-Products Compounds or Side Products

Side reactions occur during manufacturing the starting substances, materials, additives and the consumer’s food packaging use, forming various novel products. Further, the close contact between the single materials can also lead to unwanted reaction products, often unknown [67,68]. An example of neo-formed NIAS is a primary aromatic amine (PAAs) in polyurethane (PU) adhesives formed by polyols and diisocyanate monomers’ polymerization [109]. If the adhesive has not adequately cured or the ingredients have not adequately mixed, the polymerization reaction is not efficient enough, and the remaining non-polymerized aromatic isocyanates can produce PAAs in contact with water [69]. PU can often generate other by-products compounds, the cyclic adipate 1,4,7-trioxacyclotridecane-8,13-dione, likely to derive from a chemical reaction from the interaction between two common ingredients in the adhesive formula [1]. Table 3 listed scientific publications that determined by-products in different types of plastic food packaging.

### 4.3. Breakdown Products or Degradation Products

During the polymers’ manufacturer, processes like high temperatures or irradiation can cause the material’s degradation, such as UV irradiation, gamma irradiation and microwave treatment [70]. Other factors like light exposure, oxidative damage or chemicals, in general, contribute to polymer degradation, causing changes in their physicochemical properties [110]. The same way is applied for the additives, e.g., antioxidants and light stabilizers since the purpose of these substances is to act as a scavenger when oxidizing conditions such as high temperatures or microwave exposure occur, and also exposure to UV light, and their mode of action is often to be oxidized earlier than the material [70,71]. Breakdown products are almost the primary source of NIAS in the FCMs. The degradation of additives and polymers leads to novel low molecular weight substances that can migrate to food [1]. Degradation products can be further divided into the degradation of polymers and additives’ degradation [9].

Known cases of additives degradation breakdown products were reported by literature, as the degradation of common antioxidants like Irgafos 168 or Irganox 1010 results in NIAS’s appearance in the polymers [1,110]. Another example of breakdown products is the azo initiators that can degrade and form recombination products without the azo group, e.g., tetramethyl-succinonitrile 2,2′-dimethyl-2,2′-azodipropiononitrile, chlorohydrins of epoxy compounds, that are considered NIAS [7]. Table 4 listed scientific publications that determined breakdown products in different types of plastic food packaging. Most of the studies reported degradation products as NIAS, and most of the reported NIAS are degradation products from the antioxidants Irganox 1010 and Irgafos 168 [59,70,72,73,74,75,76,77,78].

### 4.4. Impurities in the Raw Materials

Raw materials and the additives may contain impurities, some of them known by the manufacturers, but minor impurities often are unknown and can persist in the final FCMs, consequently migrating to the foodstuffs [69,111]. Ink raw materials can be unique chemical substances or mixtures of many chemical substances. They comprise one or several main substances with a specific function in ink (IAS); besides these main components, the raw materials may contain other substances that will not have any specific function in ink, e.g., monomers or residues of catalysts, solvents or defoamers. Such substances are necessary to produce the raw material, but they are not needed in the printing inks. Nevertheless, such impurities are usually known due to the raw materials production process and should be specified in the supply chain to allow a risk assessment, as they should be considered “known NIAS” in the inks. However, it is not always possible to list and consider all impurities during the authorization.

An example is primary aromatic amines (PAAs) and β-naphthol in azo-pigments made for printing inks. Both substances can be present as impurities in the pigment and the final ink formulation. The azo-pigment itself is an IAS used to formulate the ink, but PAAs and β-naphthol or β-naphthol-derivates are NIAS. Another example is impurities from acrylic adhesive additives in migration tests of multilayer materials [112], Table 5 listed scientific publications that determined impurities in different types of plastic food packaging.

### 4.5. Contaminants

There are multiple sources of contaminant residues in the supply chains, including presses (fountain solutions, lubricants and additives), substrates (plasticizers, surfactants, stabilizers, antioxidants, resins), inks and coatings (resins, polymers, adhesives, pigments, solvents, monomers, additives) and the environment (pesticides, cleaners, fumes) [12]. Toxic elements such as As, Cd, Hg, Pb are trace elements and environmental contaminants present in the raw materials and considered NIAS in the final FCA. When recycled materials are utilized for food packaging, often undefined mixtures of chemicals present during recycling can react and form additional substances that increase the list of potential NIAS. Furthermore, the accumulation of chemicals might occur when materials are recycled many times. Thus, the prediction, identification and control of NIAS in recycled materials are challenging because of their difficulty tracing their origin [1,31]. Packaging materials produced from recycled polyethylene terephthalate are applied for direct food contact in recycled rigid containers and films. Most recycled polyethylene terephthalate packaging materials contain metal catalysts, the most common antimony [31]. In other cases, contaminants have been associated mainly with printing inks, paperboards, adhesives or substances added to color the final material [77,79] reported 134 substances, including volatile and semi-volatile compounds, in recycled LDPE and HDPE from domestic waste. The main groups were contamination from external sources, additives and additives breakdown products. Table 6 listed a few scientific publications that determined contaminants in different types of plastic food packaging, most of them recycled materials.

## 5. The Challenges of the NIAS Assessment

Substances with a molecular weight of more than 1000 Da are not usually considered in NIAS determination, while non-listed compounds <1000 Da are considered NIAS since substances with a higher molecular weight are not absorbed in the metabolism. An exception is applied for fluorochemicals, for which a cut-off of 1500 Da has been suggested [8], since these substances tend to have smaller molecular volumes at the same molecular mass, enabling resorption and render them potentially toxicologically relevant. However, substances migrating from FCMs may be of considerably high Mw. They may include intentionally added polymeric additives, such as polyadipates [113], and NIAS, such as oligomers, which can be formed due to an incomplete polymerization result in a later degradation of the base polymer [105]. A molecular size cut-off is typically applied in chemical risk assessment of FCMs in Europe and the US: chemicals larger than 1000 Da is not assessed. [114] published a review that pointed to the existence of FCCs with an increased intestinal permeability; this may lead to the uptake of the compound of not only low (<1000 Da) but also high (>1000 Da) molecular weight. The authors discuss the toxicological relevance of high molecular weight compounds in the gut, suggesting that the scientific justification for implementing a molecular weight-based cut-off in the risk assessment of FCMs should be reevaluated.

### 5.1. NIAS Identification: How to Start?

**Figure 4:**
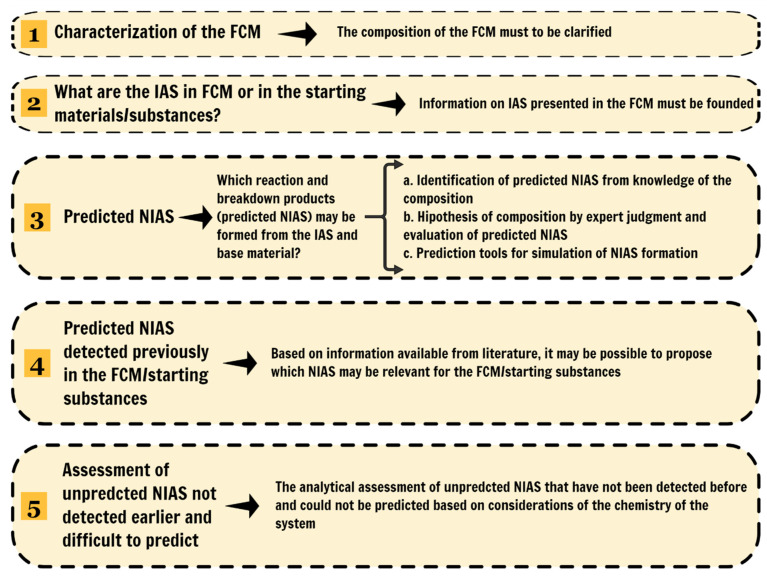
Steps approached by trying to identify predicted/unpredicted NIAS.

**Figure 5:**
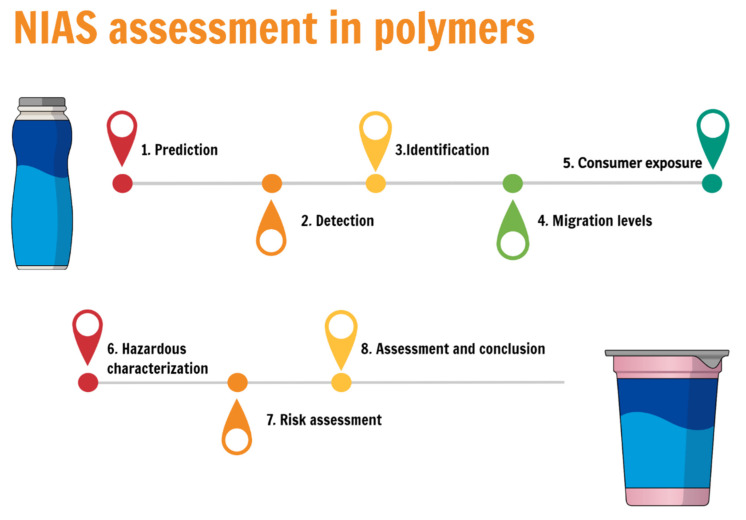
Steps involved in the NIAS assessment in polymers.

Figure 4 reports the steps detailed by Koster et al. [7] about NIAS information collection and identification in FCMs. Figure 5 shows the usual steps involved in the NIAS assessment. According to [7], since the polymer can be a mixture of different substances, detailed description and knowledge of the starting substances and processes enormously facilitates NIAS analysis in the final product. An adequate characterization of the FCM is of utmost importance for defining what type of NIAS may be expected and which analytical techniques should be used to determine migration. This includes a complete characterization of the IAS present in the FCM, the manufacturing processes, the temperature of exposure during the production, etc.

After that, the next step is to identify the predicted NIAS from the IAS knowledge present in the FCM, e.g., reaction/breakdown products formed from the IAS and base materials. Moreover, the guidance strongly recommends the literature survey to predict NIAS’s formation in each FCM and communicate with subject matter laboratories. For predicted NIAS (known NIAS), targeted analytical methods are applied, detailed in the next section.

Finally, the identification of unpredicted NIAS formed in the final FCM with potential migration to the foodstuffs was not predicted based on the system’s chemistry considerations. For example, these NIAS can be contaminants and reaction products present in products made by complex chemistry such as coatings, rubbers and adhesives. Non-targeted screening analytical approaches should be used to assess these unpredicted NIAS, detailed in the next section [7].

EFSA published in 2016 a public scientific opinion about recent developments in the risk assessment of chemicals in food and their prospective impact on the safety assessment of substances used in FCMs [8]. As the EFSA document is the most recent European official publication on the subject, emphasis will be given to this approach. The main points addressed by the document are listed below:

- although information about the substance’s identity as used and its impurities is necessary, more focus on the migration potential from finished materials and articles is needed. Substances used to manufacture FCM may disappear, and it may be mainly reaction products that turn up in the migrates;
- it is necessary to describe the chemical and the physical properties of a substance that are the determinants for its potential to persist or react in the final FCM and food and migration. The needed information includes: the volatility and thermal/chemical stability of the substances used as well as their impurities; the octanol-water partition coefficient (log Po/w) and the solubility of the migrating substances in solvents of different polarity and food and food simulants; their stability in food simulants and food hydrolysis in the gastrointestinal tract; possible chemical interactions with the food, leading to the generation of reaction products with or from the food;
- information about the level of use, the function of the substance and the manufacturing process conditions are needed for assessing the quantities, types, and nature of potentially migrating substances. Depending on the details of information accessible, such as the nature of the plastics produced using the substance, the food characteristic the plastic materials intended to contact, and whether the FCM is intended for single or repeat use applications, a more or less refined exposure estimate may be derived.

#### 5.1.1. NIAS Extraction

Currently, there is no standard protocol about how to proceed with the NIAS identification/quantification. Firstly, it is necessary to decide if the analysis will be made on the packaging material itself, on the food simulant during migration tests or direct in the food in contact with the packaging [1,115]. The most recent scientific opinion published by EFSA (2016) [8] brings three main approaches: modelling, migration and direct food measurements. In the first case, the FCM could be analyzed to identify/quantify the NIAS, and mathematical models can estimate the total mass transferred from the FCM to the foodstuff, which should be validated. The document EUR 24,514 EN 2010 (applicability of generally recognized diffusion models for estimating specific migration in support of EU Directive 2002/72/E.C.) must be consulted in this kind of approach.

In the simulation of migration, according to the recommendation of EU n° 10/2011, some food simulants (see Table 7) could be placed in contact with the FCM and posteriorly analyzed instead of the real food, with temperature conditions and contact period predetermined in the current legislation. According to the scientific opinion by EFSA (2016) [8], the EC guidelines on migration testing are under development. Finally, direct measurements in foods are needed when the simulation is impossible or not reliable. This type of approach has recently been the most used in NIAS identification/migration studies by the scientific community in different plastics FCMs [6,80,81,116]. Food simulants such as Tenax [32,68,72,73,76,83,117], water or organic solvents [25,28,31,63,69,74,83,84,85,86,87,88,108] have been used to simulate the migration of NIAS from plastic food packaging. Table 1 identifies the simulants or foodstuffs used in several studies on NIAS assessment. Some authors reported migration studies direct in food matrices, e.g., pizza [89], sliced fresh chicken breasts [87], baby food: a mixture of fruit purées (apple, banana, pear), fruit jelly, chocolate custard [66]. It is also essential to define temperature and exposure time to simulate real conditions [69].

Another challenge is sample preparation. As chromatographic techniques are the most commonly used in NIAS identification studies, any chromatographic analysis of solid samples needs an extraction or migration step that transfers as many compounds as possible into the liquid or gaseous phase or representative for what may migrate into food [84]. Organic molecules are extracted from polymeric materials by applying a solvent in which they are soluble. Different extraction techniques for organic compounds in polymers, including NIAS, have been reported in the literature, such as liquid–liquid extraction (LLE) [81,118], Solid Phase Extraction (SPE) [30,69], dispersive liquid–liquid microextraction (DLLME) [119], ultrasonic extraction [10,90,120], headspace solid-phase microextraction (HS-SPME) [73,79,91,121], solid-phase microextraction (SPME) [67,91] fabric phase sorptive extraction (FPSE) [92], accelerated solvent extraction (ASE) [74] and QuEChERS [115]. When the determination is performed in a solution, most analytical procedures involve concentration steps before the instrumental analysis [1]. Sanchis et al. [115] recently published an extensive review of relevant conventional and novelty analytical procedures for the three matrices (simulants, FCM or food) proposed in recent literature. Nerin et al. [1] cite the possibility of direct analysis in polymers by using ASAP (atmospheric solid analysis probe), DART (direct analysis in real-time) [93,108] or DESI (desorption electrospray ionization) [117], which can be done directly using high-resolution mass spectrometry (HRMS) instruments [64,68,116,122]. These methods do not require extraction steps and do not separate the analytes. Therefore, it is a quick technique but should only analyze well-known substances due to the complicated fragmentation patterns usually obtained.

For the analysis of predicted or unpredicted NIAS, two strategies must be applied: targeted analytical methods for predicting NIAS or non-targeted or screening methods to analyze substances with a wide range of physical/chemical properties [9,94]. All analysis strategies should detect and quantify the amount of NIAS present in the FCM. This is possible for predicted and known NIAS but difficult for unpredicted NIAS since reference standards may not be available. As a practical standard, the migration level of 10 μg/kg food for NIAS is applied, as this is the level from which each migrated substance must be identified [9]. This level has been specified in the Plastics Regulation (EU) No 10/2011 for migration through a functional barrier: unauthorized but intentionally added substances may be used in FCM plastics behind a functional barrier provided they do not migrate at levels above 10 μg/kg food; substances that are carcinogenic, mutagenic or toxic for reproduction (CMR) may not be used. The threshold of 10 μg/kg is a practical limit and not based on current toxicological understanding.

Usually, NIAS’s identification requires susceptible advanced analytical techniques and databases and software tools [5,9]. The general workflow for the analysis of FCMs involves the data acquisition after the selection of one or a combination of analytical techniques, such as gas chromatography (GC) for volatile and semi-volatile compounds with low polarity [78,95,123,124,125] and liquid chromatography (LC) for thermally unstable and non-volatile compounds; both coupled with mass spectrometry detectors (MS) [28,96,126].

**Figure 6:**
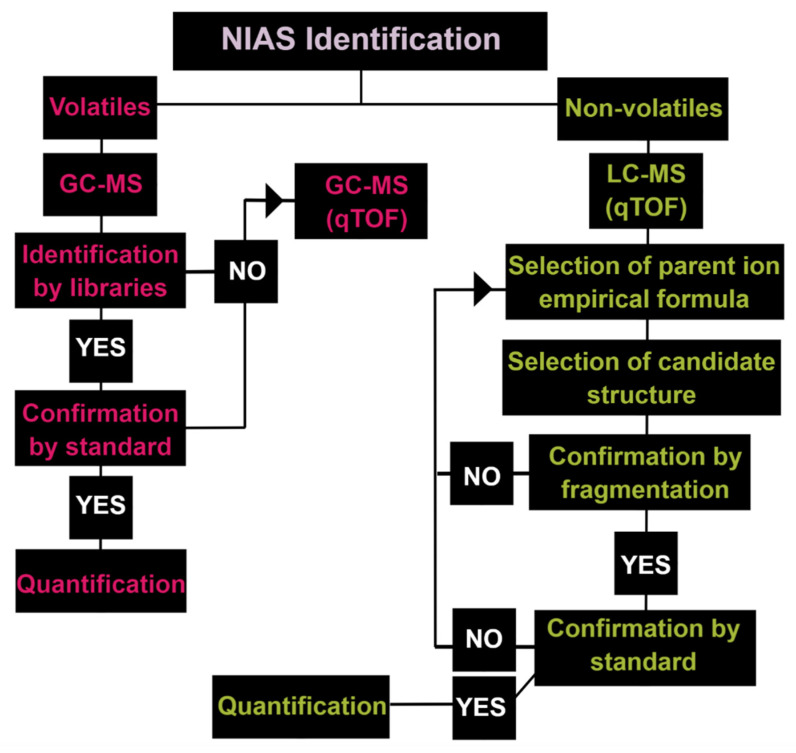
Example of decision tree for NIAS identification/quantification (adapted from Nerin et al. [1]).

The GC-MS enables mass spectral libraries for the identification (target methods) for volatile and semi-volatile compounds. For a qualitative analysis of unknown samples, GC-MS is operated in a scan mode to obtain the entire mass spectrum within the predefined peak identification mass range [4,32]. When the identification is not possible, high-resolution tandem quadrupole and time-of-flight mass analyzers (Q-TOF-MS) help identify sum formulas of unknowns (non-target methods) [33,66,69,77,82,85,86,93,127]. For non-volatile compounds, because of the absence of extensive libraries for mass spectra, the use of a Q-TOF-MS will allow accurate mass determinations of the precursor ion and the productions, providing information about fragmentation patterns. This represents excellent structural information and assures the correct identification of unknown compounds [1]. Figure 6 illustrates a decision tree for each analytical procedure to choose from NIAS identification/quantification published by [1]. Table 1 listed the most techniques utilized to determine unknown compounds in plastic food packaging, including NIAS.

#### 5.1.2. Target Analysis for Predict NIAS

An internal standard should be used for this evaluation: the same or structurally very similar (isotope-labelled) compared to the NIAS under investigation. This ensures that the detector response for the internal standard and NIAS is the same or very similar. Migrates may be obtained using migration conditions simulating the intended use of the FCM. One or more internal standards should be added at a level in the range of the NIAS’s regular migration [7].

GC-MS is the most frequently used technique for volatile, and semi-volatile knew organic compounds since the commercially available mass spectra library (e.g., NIST library) could help identify the compounds [32,78,80,123]. The libraries contain the MS spectra obtained by electronic impact and a quadrupole mass analyzer. An ion trap allows further fragmentation to a select mass fragment, which could better identify the compound. If the identification is not possible, the use of high-resolution mass spectrometry (HRMS) techniques such as time of flight (TOF) or Orbitrap instruments provides accurate mass measurements and full-scan spectra that will help [4,116]. The HRMS detectors can be accoupled to both LC and GC systems [1].

For non-volatile compounds, the previous separation in liquid chromatography of the compounds present in the sample is essential for proper identification. Even though many detectors can be coupled to LC, most of them, such as UV, DAD [29], fluorescence or IR, do not provide enough information for NIAS identification, and they are more frequently used for confirmation purposes. Liquid chromatography (LC) coupled to mass spectrometry (MS) is probably the most powerful technique for analyzing non-volatile NIAS [60,63,64]. Because of the absence of extensive libraries for mass spectra acquired by LC-MS techniques, the identification process is more complicated and time-consuming than for mass spectra obtained by GC–MS [128].

As in GC, different mass analyzers can be used in LC-MS. These include quadrupole (Q) and ion trap (IT), or high resolution (HR) mass analyzers such as time of flight (TOF) and Orbitrap [65,66,97,98]. The quadrupole is frequently used for quantitative purposes due to its high sensitivity and selectivity. However, its identification capabilities are deficient due to its reduced sensitivity in full scan mode and mass accuracy. The main benefit of HRMS techniques is the possibility of collecting full scan spectra with very accurate mass measurements, which allows the analyst to perform structural elucidations of unknown or suspected compounds [1].

#### 5.1.3. Non-Target Analysis for Unpredicted NIAS

Screening analysis can be conducted for unpredicted NIAS and detects predicted NIAS and IAS [9]. In this approach, an FCM or a starting substance(s) is extracted with one or more simulant/extraction solvents followed by analysis using several analytical methods to provide maximum coverage for all substances possible, e.g., headspace/solid-phase microextraction (SPME) [34], gas chromatography flame ionization detection (GC-FID) or gas chromatography-mass spectrometry (GC-MS) to detect volatile substances [77,91]; GC-FID or GC-MS for semi-volatile substances [77,129]; liquid chromatography ultraviolet detection (LC-UV) or LC high-resolution MS for non-volatile and polar compounds [25,28,60,78]; inductively coupled plasma (ICP-MS) for trace elements [99]; and nuclear magnetic resonance (NMR) for general screening [63,118]. High-resolution GC-MS may also be useful for the analysis of NIAS [7]. NIAS with a molecular weight exceeding 1000 Da is generally not considered relevant for risk assessment. This cut-off does not cover fluorinated substances (the cut-off for these substances is 1500 Da due to the smaller molecular diameter [8]. The emerging scientific consensus points towards the potential significance of substances above 1000 kDa for long-term chronic health effects [114].

Recently, a review published by Martínez-Bueno et al. [4] presents an overview of current analytical approaches based on HRMS analysis to identify unknown migrants (volatile and non-volatile compounds) plastic food packaging materials. The conclusions are that despite the complexity of identifying unknown migrants from plastic FCMs, recent advances in mass spectrometry techniques based on accurate mass measurements (HRMS) combined with improvements in hardware and software performance have contributed to facilitating this analytical process. According to the study, regarding separation techniques, a clear trend towards applying HRMS in LC for non-targeted analysis has been observed during the last years, being ESI (in positive mode) the most widely used ionization source. In the case of GC analysis, E. and APCI have been equally applied in non-targeted approaches. Two types of high-resolution mass analyzers have been used to identify non-targeted compounds released from plastic FCMs: quadrupole time-of-flight (Q-TOF) [25,33,63,66,69,71,72,82,85,86,87,93,96,98,100,127] and quadrupole-Orbitrap (Q-Orbitrap) [84,95,96,101]. To date, the Q-TOF detector has been used more often than Q-Orbitrap in the plastic FCMs field since the use of hybrid instruments provides more information about fragmentation patterns than their simple versions (TOF and Orbitrap). This represents additional structural information and ensures the correct identification of unknown compounds. Moreover, Orbitrap technology is more expensive than Q-TOF, leading to the increased application of the last one [4].

The main challenge of a non-targeted approach is the high number of signals generated by the HRMS instrument, making it necessary to group the number of relevant peaks. The authors suggested different data reduction techniques in non-target analysis, as a case-control comparison, intensity threshold signal to noise threshold, mass range restrictions, and homemade databases. The most used analytical strategy has been the case-control comparison, or comparing the chromatograms from control or blanks and sample material solutions as pre-processing data treatment. However, combining more than one approach was often necessary to reduce the search space’s size to manageable levels. Identification of unknown migrants by GC(EI) HRMS is usually easier than LC-HRMS due to using large mass spectral databases that support the identification [4].

Wang et al. [10] studied the identification of chemicals in a commercial PVC/PE film using a UPLC-QTOF/MS method. With this robust technique, many chemicals were identified, and some recognized NIAS from diverse sources. Six additives were selected, and their migration behaviors were simultaneously assessed. The authors concluded that investigation of migration behaviors of chemicals based on the identification results obtained by the HRMS technique could be a more practical approach for the safety evaluation of commercial packaging materials. They also mentioned the necessity of quantifying chemicals (including NIAS), whose standard substances are not available. Solving this problem requires a proper selection of reference standards that can represent the target chemicals. Another aspect is identifying chemicals in some consumable materials (syringe, syringe filter, plastic tube, etc.) used for sample pretreatment. These materials may contribute to additives also present in packaging materials, resulting in blank interference.

## 6. The NIAS Risk Assessment Challenge

The traditional approach to verify the safety of chemicals in food is to perform a specific risk assessment (RA) for each chemical. There are often data available from prior research or accredited methods for the commonly investigated IAS, but there is rarely relevant data for the more elusive NIAS. Most NIAS do not have assigned chemical structures, concentration data or characterization of hazards, and few methods can obtain these data for a large group of chemicals [130]. In the EU, FCCs that are subject to authorization include IAS (e.g., starting substances and additives used in the production of food contact plastics), and in the US, these comprehend “indirect food additives”, which are substances that come into contact with food and they are transferred into food but are not intended to be added to food [11]. In both the EU and the US, the specific requirements for toxicological testing of FCCs requiring authorization depend on estimated consumer exposures, which FCM manufacturers determine before marketing [11,21].

**Figure 7:**
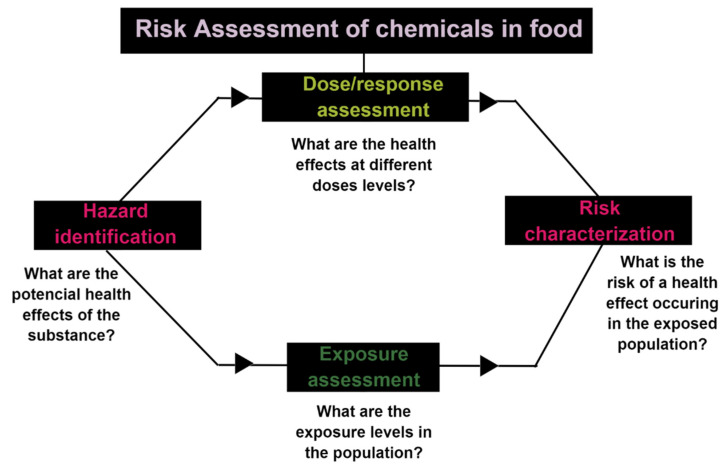
The regular approach to perform a specific risk assessment of chemicals in food (adapted from Pieke et al. [130]).

Generally, risk assessment is considered a science-based process consisting of four steps: hazard identification, dose–response assessment, exposure assessment, and risk assessment. Figure 7 shows the traditional approach to perform a specific risk assessment of food chemicals [130]. Hazard typically refers to the intrinsic properties, such as toxicity, while exposure addresses the likelihood to which a human or environmental receptor will be exposed to the intrinsic hazards. Risk is the likelihood of harm occurring. Captured into a formula, this would be: hazard x exposure potential = risk.

### 6.1. Strategies to Hazard Characterisation of NIAS

Hazard identification aims to recognize the potential adverse health effects in humans associated with exposure to a chemical. Hazard identification requires an adequate and documented review of relevant scientific information obtained from appropriate databases, peer-reviewed literature or study reports, if available. This approach emphasizes studies in the following order: human epidemiological and safety studies, animal toxicological studies, in-vitro bioassays and read-across and (quantitative) structure-activity relationships ((Q)SAR) [7].

According to the classical approach, any NIAS should go through a toxicological evaluation requiring the same toxicity data as IAS. Toxicity data of substances may be collected from existing scientific information and complemented by further in vivo, in vitro or in silico tests. However, this concept is only applicable to identified NIAS. NIAS with a known chemical structure and no toxicological data, in silico tools, may provide qualitative and/or quantitative hazard information. For example, quantitative structure-activity relationships (QSAR) allow the quantitative prediction of toxicological endpoints [80].

#### 6.1.1. Bioassays

To complement the classical approach of detecting, identifying and assessing a single NIAS, the overall migration or extract of an FCM can be tested through in vitro bioassays [25]. This approach’s interest has increased in recent years since the European regulation on chemicals (REACH) recommends that the RA of chemicals priorities in silico and in vitro methods [131]. Numerous bioassays allowing the detection of a range of bioactivities are available. Most are based on different cell types and use various biological detection principles such as transcriptional activation and cell proliferation [132]. Such tests may help detect the cumulative effects of chemical mixtures for toxicological endpoints sensitive to mixture toxicity. Extracts or migrates generating positive bioassays’ responses may subsequently be fractionated and re-analyzed to identify the active substances. In vitro bioassays offer a robust and economical solution to screen the toxicity of FCMs and FCAs and assess the overall migrates’ hazards. The results of in vitro tests could be used to highlight those FCMs or components of FCAs that are critical in terms of toxicological hazard, thus initiating a process for substituting chemicals of concern with more benign compounds. Alternatively, in vitro tests could be used to priorities samples for further testing to evaluate their potential to cause toxicity in vivo and provide input data for a whole risk assessment process [133]. However, the variability of available assays and sample preparation protocols request further optimization and standardization before bioassays can be used routinely [131] beyond the method’s complete validation. The approach results in many peaks that require identification that can be time-consuming and expensive to perform. Also, it is not comprehensible what the predictability in terms of the safety of individual peaks determined by the screening is [134].

Veyrand et al. [135] describe an initial attempt to investigate bioassays’ potential role in detecting toxicologically relevant molecules in migration simulation studies. For that purpose, the authors used as a case study a plastic cup containing the antioxidant TNPP well documented to degrade into isomers of 4NP. This chemical is known to interfere with the endocrine system, acting through activation and inhibition of the estrogen and the androgen receptors. The authors conclude that, together with analytics, bioassays contribute to identify toxicologically relevant molecules leaching from FCM and enable improved risk assessment. Ubeda et al. [25] investigated two cyclic esters’ migration from multilayer packaging material based on PU adhesive and evaluated their bioaccessibility to the body using digestion assays (in vitro). The potential formation of new compounds during gastrointestinal digestion was also evaluated. The authors observed that most samples’ oligomers’ migration values exceeded 10 ng g−1; however, bioaccessibility studies showed decreased oligomers after digestions.

#### 6.1.2. Threshold of Toxicological Concern (TTC)

The Threshold of Toxicological Concern (TTC) concept ascribes human exposure thresholds to substances with unknown toxicity and known structure [38,100]. That may be applied to evaluate materials for their potential toxicity when exposure is shallow. In the absence of toxicological data, the TTC concept is a practical risk assessment tool that sets up human exposure levels to substances considered to have no appreciable risk to human health. The TTC is currently accepted for use in the EU and the USA [4]. An Expert Group of the International Life Sciences Institute (ILSI Europe) has examined the TTC principle for its broader applicability in food safety evaluation, and they have concluded that this approach can be applied for low concentrations of chemicals in the food that lack toxicity data, provided that there is an intake estimate [7]. The authors used the TTC approach to estimate NIAS Risk Assessment in recent literature [116,136,137,138].

According to its molecular structure, the TTC principle is based on the Cramer rules, which estimate compounds’ theoretical toxicity. There are three classes of toxicity according to these rules: low (class I), moderate (class II) and high (class III). Cramer recommends a maximum intake value for each toxicity group; values for class I, II and III are 30 µg/kg bw per day, 9 µg/kg bw per day and 1.5 µg/kg bw per day, respectively [38]. The software Toxtree developed by the JRC commissioned is an open-source application that can estimate toxic hazards by applying a decision tree approach following Cramer rules (Ref http://toxtree.sourceforge.net/. Accessed on 25 May 2021). This software can be used to estimate the theoretical toxicity of the identified compounds [4].

**Figure 8:**
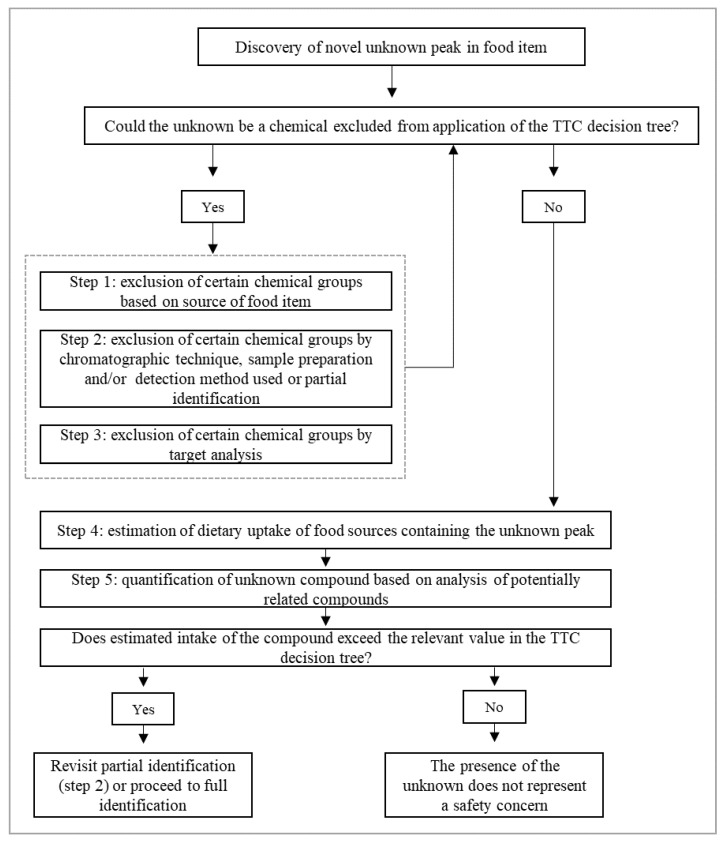
Overview of the approach to applying the Threshold of Toxicological Concern (TTC) concept to unknown peaks (adapted from Koster et al. [139]).

In 2011, Koster et al. [139] proposed a TTC approach to regulating unknown substances found in food samples, including NIAS, but NIAS’s confident identification and, particularly, genotoxic substances remain an unresolved issue [139]. Genotoxicity is a term referring to the capability of chemicals to damage genetic material. Many molecular mechanisms resulting in genotoxicity have been described, such as direct covalent binding to DNA, DNA cross-linking, DNA breakage DNA intercalation, oxidative stress and inhibition topoisomerases and interference with DNA repair [132]. Figure 8 shows the stepwise approach suggested for NIAS’s evaluation [139].

In 2012 the European Food Safety Authority (EFSA) published a scientific opinion on investigating options for providing advice about potential human health risks based on the concept of Threshold of Toxicological Concern (TTC). In 2019, EFSA published Guidance on using the TTC approach in food safety assessment. This Guidance provides clear step-by-step instructions for using the TTC approach, which can be used when the substance’s chemical structure is known, there are limited chemical-specific toxicity data, and the exposure can be estimated. According to the document, the TTC approach should not be applied for substances for which EU food/feed legislation claims the submission of toxicity data or when enough data are accessible for a RA or if the substance under consideration consists into one of the exclusion categories. For substances that can potentially be DNA-reactive mutagens or carcinogens based on the weight of evidence, the TTC value is 0.0025 µg/kg body weight (bw) per day. For organophosphates or carbamates, the TTC value is 0.3 µg/kg bw per day. All other substances are classified according to the Cramer classification.

In 2014, Koster et al. [134] proposed the Complex Mixture Safety Assessment Strategy (CoMSAS) to assess unknown NIAS’s safety in cartoon food contact material. The basis of CoMSAS is the decision tree-based approach of the application of the TTC concept [134]. This strategy is unnecessary to identify the unknown substances present in the migration extract below 90 µg/person/day, corresponding to the Cramer class III TTC threshold; substances with a structural alert for genotoxicity should be excluded a TTC threshold of 0.15 µg/day applies. The strategy respects all decisions introduced in the original TTC concept [134,140]. Several exclusion steps are introduced to exclude highly toxic substances and other substances that are excluded from applying the TTC concept [4]. In 2018, Pieke et al. [130] suggested applying quantitative structure-activity relationships (QSAR) models for carcinogenicity, mutagenicity, and reproductive toxicity as a strategy for risk prioritization based on tentative data. Four risk assessors assess a selection of 60 chemical compounds from two FCMs to classify compounds based on potential risk. For almost 60% of cases, the assessors classified compounds as either high or low priority. Unclassified compounds are due to the lack of agreement between experts or a perceived lack of data. In the high priority group, substances are high-concentration compounds, benzophenone derivatives and dyes. The low priority compounds contained, e.g., oligomers from plasticizers and linear alkane amides. The classification provides valuable information based on tentative data and prioritizes discovered chemical compounds for pending risk assessment.

In 2019, Schilter et al. [132] published an extensive review about applying the TTC to prioritize unidentified chemicals in food contact materials. The authors concluded that for a specific migrate, the evidence for the absence of mutagenicity based on the Ames test (genotoxicity testing) and analytical chemistry and information on packaging manufacture could allow the application of the Cramer class III TTC to prioritize unknown NIAS.

The Food Contact Additives guidelines (2016) on Risk Assessment of NLS and NIAS mentions the toxicological assessment, with the proposition to identify the adverse toxicological effects that a substance could cause and to define the critical dose of a substance in the daily diet, below which the substance is not expected to pose a risk to human health (dose–response assessment or hazard characterization). The critical dietary exposure level is referred to as the Tolerable Daily Intake (TDI), generally applied for substances detected in food but not intentionally added, or the Acceptable Daily Intake (ADI) for substances intentionally added to food, expressed in mg/person/day or mg/kg body weight/day. The TDI concept assumes that a clear dose–response relationship with a threshold exists, whereas the Threshold determines the point of exposure below which no adverse effect is observable. A TDI or ADI presupposes a complete in vivo study. In Europe, The Joint FAO/WHO Expert Committee on Food Additives-JECFA uses PTWI, or provisional tolerable daily intake, for contaminants accumulated in the body. The weekly designation stresses the importance of limiting intake over a while for such substances. When using this approach, no-observed-effect levels (NOELs) or no-observed-adverse-effect levels (NOAELs) are identified in the critical studies, to which appropriate safety or uncertainty factors are applied [141].

It is essential to know that two options can be considered to set a migration level above which a safety assessment of NIAS is needed: detection above the detection limit of the analytical method or detection above a level corresponding to a safe exposure threshold, the so-called exposure-based approach. In the first option, 10 µg/kg food is the conventional European limit of detection. This value has no relation to a health-derived threshold but was introduced in the Regulation (EU) n° 10/2011 as a typical detection limit of the analytical techniques used (EU, 10/2011). This 10 µg/kg threshold, although widely used for the NIAS assessment, could represent a challenge with some FCM as they may release many NIAS exceeding this limit that is sometimes difficult or not possible to identify; in the second option, a level of migration corresponding to a safe exposure threshold (based on substance-specific data or in-silico tools) could be derived. This level is also called the Level of Interest (LOI) [7]. In the US, industry and law regulatory use a similar approach, working as consultants and performing substance evaluations for food contact applications. Due to this approach, an analytical sensitivity level of 50 µg/kg, a finding of “non-detected” is found to be reasonable. In conclusion, if it can be shown that the migration of a substance, which is not CMR classified (CMR Substance: a substance listed as carcinogenic, mutagenic or toxic to reproduction category 1A, 1B or 2 in CLP Regulation 1272/2008 Annex VI Table 3.1) and shows no genotoxic structural alerts, is less than 0.01–0.05 mg/kg food (simulant), then no further assessment is needed. However, these conservative approaches have minimal applicability due to the low values (EU: 10 µg/kg, US: 50 µg/kg).

## 7. Alternatives to Plastics: Biopolymers and Bioplastics

There are two main types of biopolymers, also called renewable polymers: those that come from living organisms and those from renewable resources that need to be polymerized [142]. Biopolymers are not new. In the 1850s, a British chemist created plastics from bio-cellulose [143]. Nevertheless, in the last two decades, the gradual replacement of synthetic polymers with biopolymer counterparts has gained significant interest, considering the ever-increasing public interest in the eco-friendly use of sustainable and safe materials [144]. With a production volume of 2.11 million tons in 2018, their market share is meagre (1% of all plastics) but expected to increase [145].

A bioplastic is obtained from a biopolymer formed in a biological system such as polyhydroxyalkanoates (PHA’s), starch, cellulose and lignin. The biopolymers are, under most general conditions, also biodegradable. Bioplastics should be distinguished from synthetic (manufactured) bio-based plastics made from monomers originating from biological (once-living) systems, and bio-based polymers are not always biodegradable. Bioplastics are produced from a range of natural resources, among which agricultural products such as corn, cassava, flax fibers and agricultural by-products such as rice straw [146].

Thermoplastic-like starch (TPS), together with polylactic acid (PLA), is the leading research routes for the manufacturing of biodegradable materials [129]. Polysaccharides and proteins are often used as edible films and coating regarding their excellent film-forming properties, oxygen permeability (OP), similar to plastic films [147,148]. Other materials available are bio-based polyethylene (PE), poly trimethylene terephthalate (PTT), cellulose, chitosan, pectin, collagen, gelatin, caseins, zein, natural waxes, polybutylenes succinate (PBS), polyp-phenylene (PPP) and microbiological synthesized PHAs [143].

Very little is known about the chemical safety of bioplastics/biopolymers. These gaps in our knowledge are problematic because human exposure to chemicals from bioplastics and plant-based materials will increase with their increasing application [145]. Zimmermann et al. [145] extracted 43 bio-based and biodegradable products as well as their precursors, covering predominantly food contact materials made of nine material types, and characterized these extracts using in vitro bioassays and non-target high-resolution mass spectrometry. A total of 67% of the materials induced baseline toxicity, 42% induced oxidative stress, 23% induced antiandrogenic and one material induced estrogenicity. The authors tentatively identified 343 priority compounds, including monomers, oligomers, plastic additives, lubricants and non-intentionally added substances (NIAS). A comparison with conventional plastics indicates that bioplastics and plant-based materials are similarly toxic.

Specific migration of volatile compounds has been studied in two types of dishes (wheat pulp and wood) [149]. Those identified compounds considered of interest, according to existing legislation, have been quantified. The results showed that the quantified compounds are well below the specific migration limits (SML) set by the legislation, thereby showing the safety of using this biodegradable dish. Third-seven different compounds were detected in pellets and films of PLA-polyester blend samples by [150]. The results showed that new compounds from the reaction of PLA components with food simulants were present in migration solutions.

A biodegradable antioxidant active food packaging based on antioxidants from medicinal and aromatic plants incorporated into a polylactic acid matrix was designed and developed by Gavril et al. [151]. Moreover, an extensive investigation of the influence of sage and lemon balm leaves on material safety and the type of migrants was performed using migration assays. It was shown that the addition of sage and lemon balm leaves into a polylactic acid structure decreased the migration of both linear and cyclic polylactic acid oligomers, currently not legislated by European Union. Besides, total absence or decrease of migration of volatile compounds were observed when using the active films.

New bamboo-based biopolymers were evaluated by Osorio et al. [152] to ensure consumers safety. Twelve non-volatile compounds were detected in migration solutions, mainly melamine and its derivatives, coming from polymer resins from the biopolymer. Even though some of the compounds found in migration came from bamboo, such as phytosterols, most migrants came from the melamine to improve the biopolymer properties. Not only melamine but several melamine derivatives were found in migration above the limits established in European legislation. Consequently, this material does not comply with the EU legislation. The material cannot be identified as bamboo but as melamine with bamboo filler. As melamine is neither a biopolymer nor biodegradable material, promoting these kitchenware materials as bamboo can be considered fraud to consumers.

Two different biopolymer samples based on Polylactic acid (PLA) and compounds’ migration to food simulants were studied by Ubeda et al. [153]. Thirty-nine different PLA oligomers made of repeated monomer units of [LA] (C_3_H_4_O_2_) and different structures were identified. They corresponded to cyclic oligomers with [LA]n structure and two groups of linear oligomers, one with a hydroxyl group and the other one with an ethoxy group. Cyclic oligomers only appeared in the material and were not present in migration solutions. Linear oligomers HO–[LA]n–H was already present in the pellets/film, and they migrated in a higher extension to aqueous food simulants. However, linear oligomers CH3–CH2–O–[LA]n–H was not present initially in the pellets/film but were detected in migration to simulants with ethanol content. Furthermore, 5 cyclic polyester oligomers were identified in migration.

Canellas et al. [123] studied the migration of compounds coming from a compostable adhesive through different industrial biodegradable materials. Five of the 13 compounds identified were NIAS; they were neoformed compounds created by the reaction of added compounds in the adhesive. The migration of the compounds through different biodegradable materials—paper, polylactic acid, ecovio^®^ and polyvinyl alcohol—was studied. One of the migrants identified is 2,4,7,9-tetramethyl-5-decyne-4,7-diol, an intentionally added substance, and the other two were 1,6-dioxacyclododecane-7,12-dione and 1,6,13,18-tetraoxacyclotetracosane-7,12,19,24-tetraone, which were NIAS.

## 8. Conclusions

Plastic packaging used as food items is becoming increasingly complex, and the growing use of polymers is a fact in the coming years. Concerning the non-intentionally added substances (NIAS) in polymers, there are evident challenges. This review evidenced that further studies and strategies are needed in many different contexts, such as the definition of NIAS, identification and determination, the prediction, the sample preparation and validation, determination methods and methods validation, migration studies and validation and especially risk assessment trials and validation, consequently, the setting of maximum limits for the risk substances. This is a multidisciplinary field of study. Moreover, in many countries, there is no current legislation regarding NIAS. Despite the efforts and work published, NIAS will still be a much-explored theme over the years and remains a significant challenge for the scientific community and industry since the lack of information, guides and definitions still need resolutions.

Furthermore, with the high complexity of plastic packaging, it is a great challenge to predict all chemical reactions and by-products formed in the packaging and eventually migrate to the food. Some high points identified in this work are:

- it is estimated that most of the food contact material contain NIAS and most of the substances that migrate from plastic food packaging are unknown;
- several studies determining NIAS were carried out in polyurethane adhesives (PU), polyethylene terephthalate (PET), polyester coatings, polypropylene materials (PP), multilayers materials, plastic films, polyvinyl chloride (PVC), recycled materials, high-density polyethylene (HDPE) and low-density polyethylene (LDPE);
- breakdown products are almost the primary source of NIAS in plastic FCMs, most of the degradation products from antioxidants; following by side reaction products and oligomers;
- substances used to manufacture FCM may disappear, and it may be mainly reaction products that turn up in the migrates;
- the NIAS assessment in plastics FCMs is usually made by migration tests under worst-case conditions using food simulants (Tenax, organic solvents, water) and simulating the temperature and time of exposure;
- targeted analytical methods for the analysis of predicted NIAS are applied using GC-MS based methods for volatile NIAS and GC-MS and LC-MS based methods for semi- and non-volatile NIAS;
- non-targeted or screening methods to analyze unknown NIAS in plastic FCMs is mainly done using GC and LC techniques combined with QTOF mass spectrometry;
- for all concepts, better information transfer through the whole value chain would mainly facilitate the identification of unknown compounds since the entire supply chain has responsibility;
- NIAS could be present in the materials in deficient concentrations and still be a risk;
- in terms of risk assessment and prioritization for NIAS, the threshold of toxicological concern (TTC) concept is the most applied tool, by comparing the semi-quantitative concentration of the chemical compound with the estimated exposure limit;
- the combination of bioassays with sensitive analytical techniques seems to be an efficient way of identifying NIAS and their hazard to human exposure; mutagenicity based on the Ames test (genotoxicity testing), together with analytical chemistry and information on packaging manufacture, could allow the application of the Cramer class III TTC to prioritize unknown NIAS;
- oligomers up to a molar weight of 1000 Da seems to be relevant under the assumption of human gastrointestinal absorption, and this cut-off should be re-evaluated by legislation;
- currently, there is an absence of industry-wide harmonized methodology on dealing with NIAS;
- for commonly investigated IAS, there is often risk assessment data available for prior research or via accredited methods, but for the more elusive NIAS, there is rarely relevant data;
- there are only a few references outside Europe and the United States regarding NIAS in food contact materials, although other territories closely follow Europe and the United States legislation;
- the guidelines need to be urgently updated;
- biopolymers/bioplastics are currently being developed as an attempt to replace conventional plastics; however, few studies on the safety of these materials and migration of compounds to food have been carried out and published in the literature;
- the migration of NIAS from biopolymers/bioplastics reported in some studies emphasize the need to evaluate these alternative materials for hazardous compounds and NIAS and establish legislation for these specific new materials.

NIAS is still a challenge for the regulatory system, food industry, and for the scientific community, being necessary upgrading of the current legislation that meets the requirements of consumers and food producers, with the efforts of science with the publication of reliable data and commitment of the entire food production chain.

## Figures and Tables

**Table 1:** Studies published in the literature on NIAS Identification in different plastic food packaging.

Food Contact Material	NIAS	Method/Technique	Migration Tests	Reference
Polyvinyl chloride PVC/polyethylene-PE multilayer film	Antioxidants derivatives: Triester analog of 1010; Plasticizers (contain glycerol): 1-oleoyl-3-linoleoyl-rac-glycerol; Slip agents (with an amide end group): Tetracosenamide, Docosanamide, Icosanamide; Others: 2-(2-hydroxyethyl-hexadecylamino)ethyl palmitate, Bis(2-ethylhexyl) 2,2′-disulfanediyldiacetate	UPLC-QTOF/MS	Stainless-steel migration cell, water, 40% ethanol or 95% ethanol	[10]
Multilayer plastic materials (the combination of aluminium (Al), polyethylene terephthalate (PET), polyamide (PA), polypropylene (PP) and polyethylene (PE)	Cyclic esters (AA-DEG and AA-DEG-IPA-DEG)	UPLC-MS-QTOF and UHPLC-MS-QqQ	Ultrapure water, ethanol 10% and 95% ethanol	[25]
Polyethylene terephthalate (PET)	Cyclic oligomers	LC-MS	50% ethanol at 80 °C	[28]
Polyester resins, tin plate sheets coated with polyester–phenolic lacquers and corresponding press-twist-closures (equipped with plasticized polyvinyl chloride [PVC] sealings)	Polyester oligomers (cyclic oligomers, dimers, trimers and tetramers)	HPLC–DAD, HPLC–DAD/MS, GC-MS, GC–MSD and RP-HPLC–DAD/MS	Mashed infant food and two types of homemade carrot puree, Acetonitrile, 50% Etanol, 20% Etanol	[29]
Rigid thermoformed containers and films made with Recycled polyethylene terephthalate-RPET	Chromium, nickel	CP-AES	Distilled water, 5% citric acid	[31]
Multilayer food packaging materials	Printing unknown ink compounds	GC-MS	Tenax, isooctane and etoh 95% and etoh 50%	[32]
Polyurethane adhesive	1,4,7-trioxacyclotridecane-8,13-dione; 1,6-dioxacyclododecane-7,12-dione dimer; 1,4-dioxacyclotridecane-5,13-dione; 1,4,14,19-tetraoxacyclopent acosene-5,13,20,25-tetra one and 1,4-dioxacyclotridecane-5,13-dione; by-product of the curing reaction: 1,1-(Methanediyldibenzene-4,1-diyl)bis[3-(2-hydroxyethyl)urea]; 4-(7-acetoxy-5-methoxy-8,8-dimethyl-2-oxo-7,8-dihydro-2H,6H-pyrano[3,2-g]chromen-3-yl)-1,3-phenylene diace-tate; Bis[2-(diethylamino)ethyl] 4,4′-[(2-methyl-1,3propanediyl)bis(oxycarbonylimino)] dibenzoate and Bis[2-(diethylamino)ethyl]4,4′-[1,5-p entanediylbis(oxycarbonylim ino)] dibenzoate; unknown compounds	UPLC–Q-TOF/MS	Tenax	[33]
Expanded polystyrene (EPS) (recycled material)	Styrene dimmers are observed, like cis-1,2-diphenylcyclobutane, 2,4-diphenyl-1-butene, trans-1,2-diphenylcyclobutane and 1-phenyltetralin. These compounds were reported as by-products during styrene polymerization or material processing	HS-SPME-GC–MS	10% (*v*/*v*) ethanol and 3%(*w*/*v*) acetic acid	[34]
Polyethylene-PE, low-density polyethylene-LDPE and HIGH-density polyethylene-HDPE	Dibutyl amine, *N*,*N*-bis(2-hydroxyethyl)alkylamines (impurity reaction or breakdown products), *N*,*N*-bis(2-hydroxyethyl) dodecylamine, tributylphosphine, tridodecylamine, Methyl (Ralox 35), ethyl 3-(3,5-di-tert-butyl-4-hydroxyphenyl) propanoate (breakdown of Irganox 1010 or Irganox 1076), Benzenepropanoic acid, 3,5-bis(1,1-dimethylethyl)-4-hydroxy-, 1,1′-[2,2-bis(hydroxymethyl)-1,3-propanediyl] ester and benzenepropanoic acid, 3,5-bis(1,1-dimethylethyl)-4-hydroxy-, 1,1′-[2-[[3-[3,5-bis(1,1-dimethylethyl)-4-hydroxyphenyl]-1-oxopropoxy]methyl]-2-(hydroxymethyl)-1,3-propanediyl] ester (degradation of Irganox 1010), alkylamides *N*,*N*′-1,2-ethanediylbis-(breakdown or impurity products of the additive octadecanamide, *N*,*N*′-1,2-ethanediylbis), Irgafos 168 OXO (oxo-derivative of Irgafos 168), 11-eicosenamide (derived from oleamide)	UPLC IMS QTOF	Ethanol 95%, ethanol 50%, Tenax, ethanol 10% and acetic acid 3%	[59]
Empty cans with lids coated with polyester resins	Oligomers	GC–MS, HPLC-DAD/MS, UHPLC-HRMS and DART-HRMS	--	[60]
Polyester coatings based on NAH	Oligomers	LC-MS/MS and LC-TOF-MS	Acetonitrile, Water, 10% Aqueous ethanol (*v*/*v*), 50% Aqueous ethanol (*v*/*v*) and diverse foodstuffs	[63]
Polyester can coating extracts	Linear and cyclic oligomers derived from the incomplete polymerization of polyester monomers, phthalic acids and diols	HPLC-MS, HPLC-ESI MS and HPLC-HRMS/MS	95/5 etoh/water (*v*/*v*-%) solution for 4 h at 60 °C and 50/50 etoh/water (*v*/*v*-%) solution for 10 days at 60 °C	[64]
Virgin and recycled Polyethylene terephthalate-PET pellets	Cyclic and linear oligomers: TPA-EG, (TPA-EG)2 + H_2_O, (TPA-EG)2, (TPA-EG)3 + H20, (TPA-EG)3, (TPA-EG)4, and (TPA-EG)5, TPA2-EG-DEG + H_2_O, TPA2-EG-DEG, TPA3-EG2-DEG + H_2_O, TPA3-EG2-DEG, and TPA4-EG3-DEG, (TPA-DEG)2 and TPA4-EG2-DEG2	UPLC-MS-QTOF	Ethanol 10% *v*/*v*) and simulant B (acetic acid 3% *w*/*v*) as aqueous simulants and ethanol 95% *v/v* as a fat simulant	[65]
Baby food squeezes with multilayer materials (Polyethylene terephthalate-PET/aluminium-Al/polyethylene-PE)	Polyester oligomers, 29 cyclic and six linear oligomers. ε-caprolactam was tentatively identified as a heterogenic polyester oligomer combined with AA, DEG, PA and NPG; BHET and diethyl 5-(2-((2,4,5-trimethoxybenzoyl)oxy)acetamido)isophthalate, methoxyeugenol and Bis(2-methoxyethyl) sebacate	UHPLC-ESI-QTOF MS	Baby food: mixture of fruit purées (apple, banana, pear), fruit jelly, chocolate custard, acetic acid 3% (*w*/*v*) and ethanol 20% (*v*/*v*)	[66]
Polyurethane adhesives in multilayer packaging materials	Silane unknown compounds; degradation of antioxidants Irgafos and Irganox (2,6-Di-tert-butylbenzoquinone; isomer 2,5-di-tert-butylbenzoquinone; 7,9-Di-tert-butyl-1-oxaspiro(4,5)deca-6,9-diene-2,8-dione; benzenepropanoic acid, 3,5-bis(1,1-dimethylethyl)-4-hydroxy-methyl), by-product of the polyester-based urethane 91,6-Dioxacyclododecane-7,12-dione); cyclic adipate; unknown nitrogen-compounds; unknown phenolic compounds; 1,4,7-Trioxacyclotridecane-8,13-dione	HS-SPME-GC-MS	Tenax; isooctane	[67]
UV-curable varnishes over polypropylene	2-propenoic acid,1,1′-[2-[[3-[2,2-bis[[(1-oxo-2-propen-1-yl)oxy]methyl]butoxy]-1-oxopropoxy]methyl]-2-ethyl-1,3-propanediyl] ester is considered a NIAS, as it is a reaction product coming from the monomer TMPTA, 11-diethyl-7-oxo-4,6,10,12-tetraoxopentadecane-3,13-diyl diacrylate	GC-MS/Q and UHPLC-IMS/QTOF	Ethanol 95% (*v*/*v*) and Migracell^®^ migration cells	[68]
Polyethylene terephthalate (PET), oriented polyamide (OPA), cast polypropylene (CPP), polyethylene (PE) and PE/ethyl vinyl alcohol PE(EVOH)	Primary aromatic amines-PAAs (1,8-diazacyclotetradecane-2,9-dione; caprolactam; 1,8,15-triazacycloheneicosane-2,9,16-trione; 1,3-bis(isocyanatomethyl)-cyclohexane, 1-cyanodecane and 1,4-bis(isocyanatomethyl)-cyclohexane; l-leucyl-l-leucyl-l-leucine; 1,4,7,18,21-pentaoxa-11,14,25,28-tetraazacyclohentriacontane (9CI); l-leucine; l-leucyl-l-leucyl-l-leucyl-l-leucyl-; butanediamide; *N*4-hydroxy-*N*1-[(1S)-2-methyl-1-(1-pyrrolidinylcarbonyl)propyl]-2-pentyl-, (2R)-; triethylamine, naphtylethylenediamine; 1,8,15,22-tetraazacyclooctacosane-2,9,16,23-tetrone; urea; *N*-cyclohexyl, urea, *N*-cyclohexy-*N*′-methyl and 1-(cyclohexycarbonyl)piperazine); Dimethyl phthalate	UHPLC–Q-TOF/MSE	3% (*w*/*v*) acetic acid	[69]
Polypropylene random copolymer composite films	Irgafos 168 and its two degradation products, 2,4-di-tert-butylphenol (DP1) and tris (2,4-di-tert-butylphenyl) phosphate (DP2)	GC-MS	Isooctane	[70]
Polypropylene (PP)	Degradation products derived from phenolic antioxidants, impurity/reaction product/breakdown product of the additives, Family 1: Family formed with the reference structure; was formed by the compounds that had a similar structure constituted by a group 3,5-di-tert-butyl-4-hydroxyphenyl; Family 2: With glycerol molecule (glyceryl monostearate, glyceryl palmitate and glyceryl dihexadecanoate, an ester of an acid chain bonded to a glycerol molecule); Family 3: Dihydroxy alquilamines (amine bonded to two ethanol molecules and also an alkyl hydrocarbon chain); Family 4: ceramide and dihydroceramide (a family of waxy lipid molecules which are composed of sphingosine (an 18 carbon amino alcohol with an unsaturated hydrocarbon chain) and a fatty acid); Family 5: amides bonded by ethylene (degradation products from a lubricant losing C_2_H_4_); Other compounds (amides come from the impurities or degradation products from erucamide and oleamide widely used as slip agents)	UPLC-MS-QTOF	Ethanol 95% and 10%, acetic acid 3% and Tenax	[71]
Hot melt adhesives (Ethylene-vinyl acetate-EVA and amorphous polyolefin APAO enriched in propene)	Degradation of Irganox 1010: 3,5-di-tert-butyl-4-hydroxybenzaldehyde	UPLC-ESI-MS/QTOF	Tenax	[72]
Polyethylene terephthalate (PET) pellets	The degradation product of the antioxidants Irgafos 168 and Irganox 1010: 2,4-bis(1,1-dimethyl ethyl) phenol; 2-methyl-1,3-dioxolane; linear aldehydes; residual monomers: ethylene glycol (EG); thermal degradation products: toluene, ethylbenzene and xylene; phthalates (DEP, DIBP)	HS-SPME/GC-MS	--	[73]
Polypropylene (PP) films	Degradation products from Irgafos 168, Tinuvin 326 and Irganox 1076	HPLC-DAD and GC-FID–MS	Distilled water/ethanol—50/50 *v*/*v*	[74]
Plastic films (with and without printing ink) including PE: polyethene. PET: polyethylene terephthalate. PA: Polyamide. PP: Polypropylene. EVA: Ethylene-vinyl acetate.	2,4-di-tert-butylphenol and 2,6-di-tert-butyl-1,4-benzoquinone. 2,4-di-tert-butylphenol is a degradation product of Irgafos 168 while 2,6-di-tert-butyl-1,4-benzoquinone is a degradation product of antioxidants such as Irganox 1010, Irgafos 168 and Irganox PS 802	purge and trap (P&T) coupled to GC–MS	Isooctane and Tenax	[75]
Polypropylene (PP)	2,4-di-tert-butylphenol (degradation product of Irgafos 168 and Irganox^®^ 1010), tert-butyl-1-oxaspiro(4,5)deca-6-9-diene-2,8-dione (a by-product of the antioxidant Irganox 1010) and 2,6-di-tert-butyl-1,4-benzoquinone, a degradation product of antioxidants such as Irganox 1010, Irgafos 168 and Irganox PS 802	GC-MS	--	[76]
Polypropylene food storage containers	Degradation products: 2,4-di-tert-butylphenol and tris(2,4-di-tert-buthylphenyl)phosphate, methyl 3-(3,5-di-tert-butyl-4-hydroxyphenyl)propionate, a compound identified as product of degradation of Irganox 1076 and/or Irganox 1010; 2,6-di-tert-butylbenzoquinone (isooctane fraction) and 7,9-di-tert-butyl-1-oxaspiro(4,5)deca-6,9-diene-2,8-dione; different compounds have been identified as metabolites of bis-(2-ethylhexyl) phthalate (e.g., 2-ethylhexanoic acid, 2-ethylhexanol, phthalic acid, mono-2-ethylhexyl phthalate), and consequently suggested as possible degradation products of this phthalate; by-product Benzothiazole; degradation products *N*,*N*-bis-(2-hydroxyethyl)alkyl amine	GC × GC−ToF MS	3% (*w*/*v*) acetic acid, 10% (*v*/*v*) ethanol, and isooctane	[77]
Can coatings	Diisobutyl phthalate (DIBP), Degradation products formed from antioxidants (1,3-di-tert-butylbenzene and 2,4-di-tert-butylphenol degradation products from antioxidants Irgafos 168 or Irganox 1076, 2,6-di-tert-butyl-1,4-benzoquinone degradation products from antioxidants Irgafos 168 and Irganox 1010, 7,9-di-tert-butyl-1-oxaspiro(4,5)deca-6,9-diene-2,8-dione, a degradation product of Irganox 1010.	GC-MS and LC-MS/MS	--	[78]
Recycled pellets obtained from post-consumer low-density polyethylene (PC-LDPE) and high-density polyethylene (PC-HPDE)	Polymer degradation products: octanal and nonanal (aldehydes); 3-decanone, 2-undecanone, 2,2,4,4,6,8,8-heptamethylnonanone and 3-dodecanone (ketones); hexane (others); Additives degradation products: 3,5-di-tert-butyl-4-hydroxybenzaldehyde (aldehyde); 2,6-di-tert-butyl-1,4-benzoquinone and 3,5-di-tert-butyl-4-hydroxyacetophenone (ketones); methyl tetradecanoate, ethyl tetradecanoate and ethyl palmitate(esters); 7,9-di-tert-butyl-1-oxaspiro (4,5)deca-6,9-diene-2,8-dione (others); Contaminants from external sources: methyl lactate, hexyl acetate and dimethyl butanedioate, α-methylionone, 3-(4-Isopropylphenyl)-2-methylpropionaldehyde, α-amylcinnamaldehyde, (phenylmethylene)octanal and dipropylene glycol amog others (cosmetic ingredients); alkylbenzenes (breakdown products produced by the degradation of alkylbenzene sulfonates); contamination related to food: the lactones, 5-methylfurfural, furfural and methyl hexanoate (can derive from food flavors as well as from cosmetics ingredients); furfuryl alcohol, methyl pyruvate and 2-acetyl pyridine (food flavors), methyl-2-ethylhexanoate, acetic acid, propanoic acid, pyridine and dimethyl trisulfide (rotten food products), 2,6-diisopropylnaphthalene (paper labels).	GC/MS and HS-SPME-GC/MS	--	[79]
Plastic baby bibs (polyethylene vinyl acetate-PEVA, polyamide-PA and polyethylene-PE)	Azocine, octahydro-1-nitroso-(Possible NIAS from printing ink); 1,6-Dioxacyclododecane-7,12-dione (NIAS from polyurethane adhesive); 1-Propene-1,2,3-tricarboxylic acid, tributyl ester (Tributyl aconitate)	GC-MS	Artificial saliva	[80]
Polystyrene-PS cups and multilayer films	Styrene monomer and oligomers; polyester urethane-based oligomers (PU) cyclic oligomers: α-methylstyrene; 1,1-diphenyl-ethylene; 2,4-diphenyl-1-butene; trans-1,2-diphenycyclobutane; 2,4,6-triphenyl-1-hexene;	GC-MS	10% *v/v* ethanol in water and 50% *v/v* ethanol in water	[81]
Polyurethane adhesives	1,4,7-trioxacyclotridecane-8,13-dione, a lactone	UPLC-TQMS and UPLC-QTOF-MS	Tenax and 3% acetic acid	[82]
Polyvinylchloride (PVC)-coated cans	6-(4-methylphenyl)-1,2,4,5-tetrazin-3-amine and BGA (6-phenyl-1,3,5-triazine-2,4-diamine)	UHPLC-HRMS	Water and 3% acetic acid	[83]
Monolayer film with polylactic acid (PLA), polylimonene (PL) and zinc oxide nanoparticles (ZnO NPs)	Tripropylene glycol diacrylate; 10-Heneicosene; α-Tocopherol acetate; *N*, *N*-Diethyldodecanamide; *N*-[(9Z)-9-Octadecen-1-yl]acetamide; 1-Palmitoylglycerol and Glycerol stearate	ICP-MS, GC–Q-Orbitrap-MS and LC–Q-Orbitrap-MS	10% ethanol, 3% acetic acid	[84]
Flexible multilayer materials point by polyurethane (PU) layers	Polyamide oligomers; Anhydride of monomethyl succinate, 3,5-di-tert-butyl-4-hydroxybenzaldehyde; Erythritol monopalmitate; PU oligomers (cyclic esters made up of phthalic acid (PA); diethylene glycol (DEG) in combination 1:1 (PA-DEG) or 2:2 (PA-DEG-PA-DEG0); adipic acid (AA) or phthalic acid (PA); and diols such as diethylene glycol (DEG), neopentyl glycol (NPG), dipropylene glycol (DPG), dihydroxyalkyl ethers (dHAE), ethylene glycol (EG), propylene glycol (PG), butylene glycol (BD) or hexanediol (HD).	UPLC MS–QTOF	Ethanol 10% *v*/*v*, acetic acid 3% *w*/*v*, and ethanol 95% *v*/*v*	[85]
Active packaging: Polypropylene (PP); PP + green tea; PP/poly (ethylene-co-vinyl alcohol) EVOH; PP/EVOH + oregano; PP/EVOH + citral; EVOH; Polyethylene terephthalate-PET/EVOH + citral; PET/EVOH + cinnamon; PP/EVOH/PP; PP/EVOH + oregano	Degradation of active compounds; impurities from the raw materials; additives used in the manufacture of the active polymer (citral thermal reaction products; oxidation product of citral; decomposition product of adipates used as plasticizers; impurity/reaction product/breakdown product for the additives used in the manufacture of PE materials; xanthenone derivates)	UPLC-QTOF-MS	Ethanol 10%; ethanol 95%	[86]
Polyethylene terephthalate (PET) film with an acrylic resin	Ethyl lauroyl arginate (LAE) impurities: *N*2-Dodecanoyl-*L*-arginine (LAS)	UPLC–MS(QTOF)	Ethanol 10%; ethanol 95%; sliced fresh chicken breasts	[87]
Multilayer materials	1,4,7-trioxacyclotridecane-8,13-dione, and diethylene glycol (DEG) [AA-DEG].	LC-HRMS	Ethanol 95% and Tenax	[88]
Silicone moulds and teats	Side reactions in the polymerization (cyclic and linear polydimethylsiloxanes; oligomeric dimethyl siloxanes)	H-NMR and GC-MS	Pizza	[89]
Polyurethane adhesives commonly used for food-contact laminated films	No NIAS detected.	GC–MS	Isooctane	[90]
Polyvinylchloride (PVC)—and polyethylene (PE)—based cling-films	2-ethyl hexanoic acid (2-EHA), triacetin	Solid-Phase Micro-Extraction and GC/MS	PDO Italian cheeses during cold storage under light or dark	[91]
Oriented polypropylene (OPP) and polyethylene terephthalate (PET) with printing inks	Printing unknown ink compounds	UPLC-QTOF-MS	Ethanol (95%) and Tenax	[92]
Polyurethanes (PURs)	Pyridine (NIAS, solvent); Dimethylacetamide (NIAS, solvent); 1,4-Dioxane (NIAS, reaction medium); Aniline NIAS, precursor, o-Toluidine NIAS, degradation product, Diaminotoluene NIAS, intermediate, o-Anisidine NIAS, intermediate, 1,4,7-trioxacyclotridecane-8,13-dione, Myristamide NIAS, contaminant, Palmitamide NIAS, contaminant, Oleamide NIAS, contaminant, Stearamide NIAS, contaminant.	GC-MS and DART-MS	--	[93]
Polycarbonate (PC)	Oligomers; PC-degradation products	UHPLC–ESI Q-orbitrap	--	[94]
Candy wrappers based on plastic and paper materials	2,6-Di-tert-butyl-4-methylene-2,5-cyclohexadienone, a degradation product of BHT, Diethyl maleate, Triacetin, Propanoic acid, 2-methyl-, 3-Hydroxy-2,4,4-trimethylpentyl ester, Diethyl phthalate, Diisobutyl phthalate, 7,9-Di-tert-butyl-1-oxaspiro(4,5)deca-6-9-diene-2,8-dione, Heneicosane, Tributyl aconitate, Docosane, Tricosane, Tetracosane, Pentacosane, Hexacosane, Heptacosane, Octocosane, Squalene, n-Nonacosane, Glycerol tricaprylate	GC-MS	--	[95]
Polyester-polyurethane lacquers	Impurities or degradation products of IPDI trimer IPDI and DPMDI, two cyclic oligoesters, 2EG + 2TPA and 2NPG + 2oPA	GC-(EI)qMS, GC-(EI)Orbitrap, GC-(APCI)TOFHRMS and GC(×GC)-(EI)TOFLRMS	--	[96]
Polybutylene terephthalate (PBT)	Cyclic oligomers from dimer to pentamer containing TPA and BD, cyclic oligomers, linear oligomers, dehydration products	HPLC-DAD/ESI-MS	--	[97]
Multilayer plastic materials (polyethylene (PE) and low-density polyethylene (LDPE) plus nylon)	Four cyclic oligomers of caprolactam (dimer, trimer, tetramer, and pentamer); by-products-cyclic ester oligomers made of the monomers adipic acid (AA), phthalic acid (PA), diethylene glycol (DEG), monoethylene glycol (MEG) and neopentilglycol (NPG); Nylon cyclic dimer, Caprolactam Cyclic Trimer, AA-DEG, Caprolactam Cyclic Tetramer, Caprolactam Cyclic Pentamer, PA-DEG, Cyclic ester made up of Phthalic acid and diethylene glycol in combination 1:2, AA-MEG-AA-MEG, AA-MEG-AA-DEG, AA-DEG-AA-DEG, PA-MEG-AA-DEG, PA-DEG-PA-DEG, PA-DEG-AA-NPG, AA-BD, AA-BD-AA-BD, AA-DEG + H_2_O, AA-DEG-PA-DEG + H_2_O, 3,6,9,12,15-pentaoxabicyclo(15.3.1)henicosa-1(21),17,19-triene-2,16-dione, 1,6-dioxacyclodecane-7,12-dione, 1,6-dioxacyclodecane-7,12-dione, 1,6,13,18-tetraoxacyclotetracosane-2,5,14,17-tetrone	LC-HRAMS and LC-ESI-Q-TOF-MS	3% acetic acid in water (*w*/*v*) and 20% of ethanol in water (*v*/*v*)	[98]
Low-Density Polyethylene (LDPE) films	Calcite (CaCO_3_), calcium sulphate (CaSO_4_), polystyrene (PS) and titanium dioxide (TiO_2_), Ca and Ti	Raman spectroscopy and ICP-MS	--	[99]
Water-based acrylic adhesive	2-(12-(methacryloyloxy) dodecyl)malonic acid	GC-MS and UPLC-QTOF	Poly(2,6-diphenyl-p-phenylene oxide) (Tenax^®^)	[100]
High and low-density polyethylene(HDPE and LDPE)	Phthalic anhydride, phthalic acid, di-butyl phthalate (DBP) and bis(2-ethylhexyl) phthalate (DEHP)	FIA-MS, LTQ-Orbitrap	--	[101]
Multilayer materials with barrier properties	Acids (nonanoic acid), Alcohols (2-nonen-1-ol), Aldehydes (5-hydroxymethylfurfural), Aldehydes (5-hydroxymethylfurfural), Alkanes (n-dodecane), Alkenes (1-undecene), Antioxidants (2,6-di-tert-butyl-4-methylphenol), Aromatics (1,3-di-tert-butyl-benzene), Cyclics (*n*-propyl-cyclohexane), Esters (ethyl hydrogen sebacate), Ethers (1,1′-oxybis-octane), Ketones (2-undecanone), Oxidation Products (2,6-di-tert-butyl-1,4-benzoquinone)	SPME–GC–MS	--	[102]
Polyester Coatings	Oligomers	HPLC-DAD/CAD, HPLC-MS and HPLC-MS/MS	Water, 3% acetic acid, 10% ethanol, 50% ethanol, and isooctane	[103]
Low-density polyethylen-LDPE and polyamide-PA added of NBBS, α-MSD, Irganox 1081, Irganox 1222, Santonox; LDPE 2/PA 6 2: Nonox A, Neozon D, Antioxidant 2246, Tinuvin P, TOTM	Degradation products of TOTM, including DEHP, isophthalate bis(2-ethylhexyl)benzene-1,3-dicarboxylate (DOIP); decomposition product of NBBS was *N*-ethyl-*N*-methylbenzenesulfonamide; The formation of the cyclic saturated isomer (1,1,3-trimethyl-3-phenyl-2H-indene) is triggered by thermal impact, and so is the rearrangement of the carbon double bond to form isomer 2,4-diphenyl-4-methyl-2(E)-pentene). Decomposition product 2,3-dimethyl-3-phenylbutan-2-yl)benzene is formed by combining two cumyl radicals during pyrolysis of the pure additive and pyrolysis of LDPE entailing α-MSD. The degradation product of Neozon D and Nonox A identified in oxidative pyrolysis of the pure analyte was 10-methyl-benz[a]acridine; degradation products of Antioxidant 2246, Santonox, Irganox 1222/1081 and Tinuvin P: o-cresol, m-cresol or p-cresol, 2-tert-butyl-4-methylphenol and 2-tert-butyl-4,6-dimethylphenol, 3,5-di-tert-butyl-4-hydroxybenzaldehyde	Pyr-GC–MS and GC-EI-MS/MS	--	[104]

DART-HRMS: direct analysis in real-time ionization coupled with high-resolution mass spectrometry; FIA-MS: low injection analysis (FIA) mass spectrometry; GC(×GC); (EI)TOFLRMS: two-dimensional gas chromatography-electron ionization time-of-flight high-resolution mass spectrometry; GC-(APCI)TOFHRMS: gas chromatography atmospheric-pressure chemical ionization time-of-flight high-resolution mass spectrometry; GC-(EI)Orbitrap: gas chromatography/electron ionization Orbitrap; GC-(EI)qMS: gas chromatography/electron ionization-quadrupole mass spectrometry; GC × GC−ToF MS: two-dimensional gas chromatography-time-of-flight mass spectrometry; GC-FID–MS: gas chromatography-mass spectrometry and flame ionization detector; GC-MS/Q: gas chromatography-quadrupole mass spectrometry; GC-MS: gas chromatography-mass spectrometry; GC–Q-Orbitrap-MS: gas chromatography and high-resolution mass spectrometry with Orbitrap analyzer; H-NMR: Proton nuclear magnetic resonance spectroscopy; HPLC-DAD/CAD: high-performance liquid chromatography coupled with diode array detector (DAD) and charged aerosol detector (CAD); HPLC-DAD/ESI-MS: high-performance liquid chromatography coupled to photodiode array detector and mass spectrometer; HPLC-DAD/MS: high-performance liquid chromatographic-mass spectrometry with diode-array detection; HPLC-DAD: high-performance liquid chromatographic method with diode-array detection; HPLC-ESI-MS: ultra-performance liquid chromatography-electrospray ionization mass spectrometry; HPLC-HRMS/MS: high-performance liquid chromatography high-resolution mass spectrometry; HPLC-MS/MS: high-performance liquid chromatography-tandem mass spectrometry; HPLC-MS: high-performance liquid chromatography-tandem mass spectrometry; HS-SPME-GC-MS: a combination of headspace solid-phase microextraction (HS-SPME) and gas chromatography-mass spectrometry; ICP-AES: inductively coupled plasma atomic emission spectrometry; ICP-MS: inductively coupled plasma mass spectrometry; LC-ESI-Q-TOF-MS: liquid chromatography-electrospray ionization mass spectrometer quadrupole time of flight; LC-HRMS: liquid chromatography high-resolution mass spectrometry; LC-MS/MS: Liquid chromatography-tandem mass spectrometry; LC-MS: liquid chromatography-mass spectrometry; LC–Q-Orbitrap-MS: liquid chromatography and high-resolution mass spectrometry with Orbitrap analyzer; LC-TOF-MS: liquid chromatography–quadrupole time of flight mass spectrometry; LTQ-Orbitrap Linear Trap Quadropole Orbitrap; Pyr-GC–MS: Pyrolysis gas chromatography-mass spectrometry; RP-HPLC–DAD/MS: Reverse phase-high performance liquid chromatography coupled with diode array absorption and mass spectrometry; UHPLC–ESI Q-orbitrap: ultra-high-performance liquid chromatography-electrospray ionization quadrupole Orbitrap mass spectrometry; UHPLC-HRMS: ultra-performance liquid chromatography high-resolution mass spectrometry; UHPLC-MS-QqQ: ultra-high-performance liquid chromatography coupled with triple quadrupole mass spectrometry; UHPLC–Q-TOF/MSE: ultra-performance liquid chromatography coupled with a hybrid quadrupole time-of-flight mass spectrometry; UPLC IMS QTOF: ultra-performance liquid chromatography-ion mobility separation-quadruple time-of-flight; UPLC-ESI-MS/QTOF: ultra-performance liquid chromatography-electrospray ionization mass spectrometer quadrupole time of flight; UPLC-QTOF-MS; UPLC–MS(QTOF): ultra-performance liquid chromatography–quadrupole time of flight mass spectrometry; UPLC-TQMS: ultra-performance liquid chromatography-tandem quadrupole mass spectrometry.

**Table 2:** Studies published in the literature on oligomers (NIAS) Identification in different plastic food packaging.

Food Contact Material	NIAS	Method/Technique	Reference
Polycarbonate (PC)	Oligomers	UHPLC–ESI Q-orbitrap	[94]
Polyester Coatings	Oligomers	HPLC-DAD/CAD, HPLC-MS and HPLC-MS/MS	[60]
Polyester coatings based on NAH	Oligomers	LC-MS/MS and LC-TOF-MS	[63]
Polyester can coating extracts	Linear and cyclic oligomers derived from the incomplete polymerization of polyester monomers, phthalic acids and diols	HPLC-MS, HPLC-ESI MS and HPLC-HRMS/MS	[64]
Empty cans with lids coated with polyester resins	Oligomers	GC–MS, HPLC-DAD/MS, UHPLC-HRMS and DART-HRMS	[60]
Polyester resins, tin plate sheets coated with polyester–phenolic lacquers and corresponding press-twist-closures (equipped with plasticized polyvinyl chloride (PVC) sealings)	Polyester oligomers (cyclic oligomers, dimers, trimers and tetramers)	HPLC–DAD, HPLC–DAD/MS, GC-MS, GC–MSD and RP-HPLC–DAD/MS	[29]
Flexible multilayer materials point by polyurethane (PU) layers	Polyamide oligomers, PU oligomers (cyclic esters made up of phthalic acid (PA)	UPLC MS–QTOF	[85]
Polyethylene terephthalate (PET)	Cyclic oligomers	LC-MS	[28]
Virgin and recycled Polyethylene terephthalate-PET pellets	Cyclic and linear oligomers: TPA-EG, (TPA-EG)2 + H_2_O, (TPA-EG)2, (TPA-EG)3 + H20, (TPA-EG)3, (TPA-EG)4, and (TPA-EG)5, TPA2-EG-DEG + H_2_O, TPA2-EG-DEG, TPA3-EG2-DEG + H_2_O, TPA3-EG2-DEG, and TPA4-EG3-DEG, (TPA-DEG)2 and TPA4-EG2-DEG2	UPLC-MS-QTOF	[65]
Baby food squeezes with multilayer materials (Polyethylene terephthalate-PET/aluminium-Al/polyethylene-PE)	Polyester oligomers, 29 cyclic and six linear oligomers. ε-caprolactam was tentatively identified as a heterogenic polyester oligomer combined with AA, DEG, PA and NPG; BHET and diethyl 5-(2-((2,4,5-trimethoxybenzoyl)oxy)acetamido)isophthalate, methoxyeugenol and Bis(2-methoxyethyl) sebacate	UHPLC-ESI-QTOF MS	[66]
Polybutylene terephthalate (PBT)	Cyclic oligomers from dimer to pentamer containing TPA and BD, cyclic oligomers, linear oligomers, dehydration products	HPLC-DAD/ESI-MS	[97]
Multilayer plastic materials (polyethylene (PE) and low-density polyethylene (LDPE) plus nylon	Four cyclic oligomers of caprolactam (dimer, trimer, tetramer, and pentamer); by-products-cyclic ester oligomers made of the monomers adipic acid (AA), phthalic acid (PA), diethylene glycol (DEG), monoethylene glycol (MEG) and neopentilglycol (NPG); Nylon cyclic dimer, Caprolactam Cyclic Trimer, AA-DEG, Caprolactam Cyclic Tetramer, Caprolactam Cyclic Pentamer, PA-DEG, Cyclic ester made up of Phthalic acid and diethylene glycol in combination 1:2, AA-MEG-AA-MEG, AA-MEG-AA-DEG, AA-DEG-AA-DEG, PA-MEG-AA-DEG, PA-DEG-PA-DEG, PA-DEG-AA-NPG, AA-BD, AA-BD-AA-BD, AA-DEG + H_2_O, AA-DEG-PA-DEG + H_2_O, 3,6,9,12,15-pentaoxabicyclo(15.3.1)henicosa-1(21),17,19-triene-2,16-dione, 1,6-dioxacyclodecane-7,12-dione, 1,6-dioxacyclodecane-7,12-dione, 1,6,13,18-tetraoxacyclotetracosane-2,5,14,17-tetrone	LC-HRAMS and LC-ESI-Q-TOF-MS	[98]
Polystyrene-PS cups and multilayer films	Styrene monomer and oligomers; polyester urethane-based oligomers (PU) cyclic oligomers: α-methylstyrene; 1,1-diphenyl-ethylene; 2,4-diphenyl-1-butene; trans-1,2-diphenycyclobutane; 2,4,6-triphenyl-1-hexene;	GC-MS	[81]

DART-HRMS: direct analysis in real-time ionization coupled with high-resolution mass spectrometry; GC-MS: gas chromatography-mass spectrometry; HPLC-DAD/CAD: high-performance liquid chromatography coupled with diode array detector (DAD) and charged aerosol detector (CAD); HPLC-DAD/ESI-MS: high-performance liquid chromatography coupled to photodiode array detector and mass spectrometer; HPLC-DAD/MS: high-performance liquid chromatographic-mass spectrometry with diode-array detection; HPLC-ESI-MS: ultra-performance liquid chromatography-electrospray ionization mass spectrometry; HPLC-HRMS/MS: high-performance liquid chromatography high-resolution mass spectrometry; HPLC-MS/MS: high-performance liquid chromatography-tandem mass spectrometry; HPLC-MS: high-performance liquid chromatography-mass spectrometry; HPLC-MS: high-performance liquid chromatography-tandem mass spectrometry; LC-ESI-Q-TOF-MS: liquid chromatography-electrospray ionization mass spectrometer quadrupole time of flight; LC-HRMS: liquid chromatography high-resolution mass spectrometry; LC-MS/MS: Liquid chromatography-tandem mass spectrometry; LC-MS: liquid chromatography-mass spectrometry; LC-TOF-MS: liquid chromatography–quadrupole time of flight mass spectrometry; RP-HPLC–DAD/MS: Reverse phase-high performance liquid chromatography coupled with diode array absorption and mass spectrometry; UHPLC–ESI Q-orbitrap: ultra-high-performance liquid chromatography-electrospray ionization quadrupole Orbitrap mass spectrometry; UPLC-ESI-MS/QTOF: ultra-performance liquid chromatography-electrospray ionization mass spectrometer quadrupole time of flight; UPLC–MS(QTOF): ultra-performance liquid chromatography–quadrupole time of flight mass spectrometry.

**Table 3:** Studies published in the literature on by-products/side reaction products (NIAS) Identification in different plastic food packaging.

Food Contact Material	NIAS	Method/Technique	Reference
Active packaging: Polypropylene (PP); PP + green tea; PP/poly (ethylene-co-vinyl alcohol) EVOH; PP/EVOH + oregano; PP/EVOH + citral; EVOH; Polyethylene terephthalate-PET/EVOH + citral; PET/EVOH + cinnamon; PP/EVOH/PP; PP/EVOH + oregano	Degradation of active compounds; impurities from the raw materials; additives used in the manufacture of the active polymer (citral thermal reaction products; oxidation product of citral; decomposition product of adipates used as plasticizers; impurity/reaction product/breakdown product for the additives used in the manufacture of PE materials; xanthenone derivates)	UPLC-QTOF-MS	[86]
Polyurethane adhesives in multilayer packaging materials	By-product of the polyester-based urethane 91,6-Dioxacyclododecane-7,12-dione); cyclic adipate; unknown nitrogen-compounds; unknown phenolic compounds; 1,4,7-Trioxacyclotridecane-8,13-dione	HS-SPME-GC-MS	[67]
Polyethylene terephthalate (PET), oriented polyamide (OPA), cast polypropylene (CPP), polyethylene (PE) and PE/ethyl vinyl alcohol PE(EVOH)	Primary aromatic amines-PAAs (1,8-diazacyclotetradecane-2,9-dione; caprolactam; 1,8,15-triazacycloheneicosane-2,9,16-trione; 1,3-bis(isocyanatomethyl)-cyclohexane, 1-cyanodecane and 1,4-bis(isocyanatomethyl)-cyclohexane; l-leucyl-l-leucyl-l-leucine; 1,4,7,18,21-pentaoxa-11,14,25,28-tetraazacyclohentriacontane (9CI); l-leucine; l-leucyl-l-leucyl-l-leucyl-l-leucyl-; butanediamide; *N*4-hydroxy-*N*1-[(1S)-2-methyl-1-(1-pyrrolidinylcarbonyl)propyl]-2-pentyl-, (2R)-; triethylamine, naphtylethylenediamine; 1,8,15,22-tetraazacyclooctacosane-2,9,16,23-tetrone; urea; N-cyclohexyl, urea, *N*-cyclohexy-*N*′-methyl and 1-(cyclohexycarbonyl)piperazine); Dimethyl phthalate	UHPLC–Q-TOF/MSE	[69]
Silicone moulds and teats	Side reactions in the polymerization (cyclic and linear polydimethylsiloxanes; oligomeric dimethyl siloxanes)	H-NMR and GC-MS	[89]
Multilayer plastic materials (polyethylene (PE) and low-density polyethylene (LDPE) plus nylon	By-products-cyclic ester oligomers made of the monomers adipic acid (AA), phthalic acid (PA), diethylene glycol (DEG), monoethylene glycol (MEG) and neopentilglycol (NPG)	LC-HRAMS and LC-ESI-Q-TOF-MS	[98]
Polypropylene food storage containers	By-product Benzothiazole	GC × GC−ToF MS	[77]
UV-curable varnishes over polypropylene	2-propenoic acid,1,1′-[2-[[3-[2,2-bis[[(1-oxo-2-propen-1-yl)oxy]methyl]butoxy]-1-oxopropoxy]methyl]-2-ethyl-1,3-propanediyl] ester is considered a NIAS, as it is a reaction product coming from the monomer TMPTA, 11-diethyl-7-oxo-4,6,10,12-tetraoxopentadecane-3,13-diyl diacrylate	GC-MS/Q and UHPLC-IMS/QTOF	[68]

GC(×GC)-(EI)TOFLRMS: two-dimensional gas chromatography-electron ionization time-of-flight high-resolution mass spectrometry; GC-MS: gas chromatography-mass spectrometry; H-NMR: Proton nuclear magnetic resonance spectroscopy; HS-SPME-GC-MS: a combination of headspace solid-phase microextraction (HS-SPME) and gas chromatography-mass spectrometry; LC-ESI-Q-TOF-MS: liquid chromatography-electrospray ionization mass spectrometer quadrupole time of flight; LC-HRMS: liquid chromatography high-resolution mass spectrometry; UHPLC–Q-TOF/MSE: ultra-performance liquid chromatography coupled with a hybrid quadrupole time-of-flight mass spectrometry; UPLC IMS/QTOF: ultra-performance liquid chromatography-ion mobility separation-quadruple time-of-flight; UPLC-QTOF-MS; UPLC–MS(QTOF): ultra-performance liquid chromatography–quadrupole time of flight mass spectrometry.

**Table 4:** Studies published in the literature on degradation/breakdown products (NIAS) Identification in different plastic food packaging.

Food Contact Material	NIAS	Method/Technique	Reference
Active packaging: Polypropylene (PP); PP + green tea; PP/poly (ethylene-co-vinyl alcohol) EVOH; PP/EVOH + oregano; PP/EVOH + citral; EVOH; Polyethylene terephthalate-PET/EVOH + citral; PET/EVOH + cinnamon; PP/EVOH/PP; PP/EVOH + oregano	Degradation of active compounds; impurities from the raw materials; additives used in the manufacture of the active polymer (citral thermal reaction products; oxidation product of citral; decomposition product of adipates used as plasticizers; impurity/reaction product/breakdown product for the additives used in the manufacture of PE materials; xanthenone derivates)	UPLC-QTOF-MS	[86]
Polyurethane adhesives in multilayer packaging materials	Silane unknown compounds; degradation of antioxidants Irgafos and Irganox (2,6-Di-tert-butylbenzoquinone; isomer 2,5-di-tert-butylbenzoquinone; 7,9-Di-tert-butyl-1-oxaspiro(4,5)deca-6,9-diene-2,8-dione; benzenepropanoic acid, 3,5-bis(1,1-dimethylethyl)-4-hydroxy-methyl)	HS-SPME-GC-MS	[67]
Hot melt adhesives (Ethylene-vinyl acetate-EVA and amorphous polyolefin APAO enriched in propene)	Degradation of Irganox 1010: 3,5-di-tert-butyl-4-hydroxybenzaldehyde	UPLC-ESI-MS/QTOF	[72]
Polyethylene terephthalate-PET pellets	The degradation product of the antioxidants Irgafos 168 and Irganox 1010: 2,4-bis(1,1-dimethyl ethyl) phenol; 2-methyl-1,3-dioxolane; linear aldehydes; residual monomers: ethylene glycol (EG); thermal degradation products: toluene, ethylbenzene and xylene; phthalates (DEP, DIBP)	HS-SPME/GC-MS	[73]
Polycarbonate (PC)	Oligomers; PC-degradation products	UHPLC–ESI Q-orbitrap	[94]
Polypropylene (PP) films	Degradation products from Irgafos 168, Tinuvin 326 and Irganox 1076	HPLC-DAD and GC-FID–MS	[74]
Candy wrappers based on plastic and paper materials	2,6-Di-tert-butyl-4-methylene-2,5-cyclohexadienone, a degradation product of BHT, Diethyl maleate, Triacetin, Propanoic acid, 2-methyl-, 3-Hydroxy-2,4,4-trimethylpentyl ester, Diethyl phthalate, Diisobutyl phthalate, 7,9-Di-tert-butyl-1-oxaspiro(4,5)deca-6-9-diene-2,8-dione, Heneicosane, Tributyl aconitate, Docosane, Tricosane, Tetracosane, Pentacosane, Hexacosane, Heptacosane, Octocosane, Squalene, n-Nonacosane, Glycerol tricaprylate	GC-MS	[95]
Polypropylene (PP)	Degradation products derived from phenolic antioxidants, impurity/reaction product/breakdown product of the additives, Family 1: Family formed with the reference structure; was formed by the compounds that had a similar structure constituted by a group 3,5-di-tert-butyl-4-hydroxyphenyl; Family 2: With glycerol molecule (glyceryl monostearate, glyceryl palmitate and glyceryl dihexadecanoate, an ester of an acid chain bonded to a glycerol molecule); Family 3: Dihydroxy alquilamines (amine bonded to two ethanol molecules and also an alkyl hydrocarbon chain); Family 4: ceramide and dihydroceramide (a family of waxy lipid molecules which are composed of sphingosine (an 18 carbon amino alcohol with an unsaturated hydrocarbon chain) and a fatty acid); Family 5: amides bonded by ethylene (degradation products from a lubricant losing C_2_H_4_); Other compounds (amides come from the impurities or degradation products from erucamide and oleamide widely used as slip agents)	UPLC-MS-QTOF	[71]
Polypropylene (PP)	2,4-di-tert-butylphenol (degradation product of Irgafos 168 and Irganox^®^ 1010), tert-butyl-1-oxaspiro(4,5)deca-6-9-diene-2,8-dione (a by-product of the antioxidant Irganox 1010) and 2,6-di-tert-butyl-1,4-benzoquinone, a degradation product of antioxidants such as Irganox 1010, Irgafos 168 and Irganox PS 802	GC-MS	[76]
Polypropylene random copolymer composite films	Irgafos 168 and its two degradation products, 2,4-di-tert-butylphenol (DP1) and tris (2,4-di-tert-butylphenyl) phosphate (DP2)	GC-MS	[70]
Plastic films (with and without printing ink) including PE: polyethylene. PET: Polyethylene terephthalate. PA: Polyamide. PP: Polypropylene. EVA: Ethylene-vinyl acetate.	2,4-di-tert-butylphenol and 2,6-di-tert-butyl-1,4-benzoquinone. 2,4-di-tert-butylphenol is a degradation product of Irgafos 168 while 2,6-di-tert-butyl-1,4-benzoquinone is a degradation product of antioxidants such as Irganox 1010, Irgafos 168 and Irganox PS 802	purge and trap (P&T) coupled to GC–MS	[75]
Polypropylene food storage containers	Degradation products, 2,4-di-tert-butylphenol and tris(2,4-di-tert-buthylphenyl)phosphate, methyl 3-(3,5-di-tert-butyl-4-hydroxyphenyl)propionate, a compound identified as product of degradation of Irganox 1076 and/or Irganox 1010, 2,6-di-tert-butylbenzoquinone (isooctane fraction) and 7,9-di-tert-butyl-1-oxaspiro(4,5)deca-6,9-diene-2,8-dione, 2-ethylhexanoic acid, 2-ethylhexanol, phthalic acid, mono-2-ethylhexyl phthalate), and consequently suggested as possible degradation products of this phthalate, by-product Benzothiazole, degradation products *N*,*N*-bis-(2-hydroxyethyl)alkyl amine	GC × GC−ToF MS	[77]
Polyethylene-PE, low-density polyethylene-LDPE and HIGH-density polyethylene-HDPE	Dibutyl amine, *N*,*N*-bis(2-hydroxyethyl)alkylamines (impurity reaction or breakdown products), *N*,*N*-bis(2-hydroxyethyl) dodecylamine, tributylphosphine, tridodecylamine, Methyl (Ralox 35), ethyl 3-(3,5-di-tert-butyl-4-hydroxyphenyl) propanoate (breakdown of Irganox 1010 or Irganox 1076), Benzenepropanoic acid, 3,5-bis(1,1-dimethylethyl)-4-hydroxy-, 1,1′-[2,2-bis(hydroxymethyl)-1,3-propanediyl] ester and benzenepropanoic acid, 3,5-bis(1,1-dimethylethyl)-4-hydroxy-, 1,1′-[2-[[3-[3,5-bis(1,1-dimethylethyl)-4-hydroxyphenyl]-1-oxopropoxy]methyl]-2-(hydroxymethyl)-1,3-propanediyl] ester (degradation of Irganox 1010), alkylamides *N*,*N*′-1,2-ethanediylbis-(breakdown or impurity products of the additive octadecanamide, *N*,*N*′-1,2-ethanediylbis), Irgafos 168 OXO (oxo-derivative of Irgafos 168), 11-eicosenamide (derived from oleamide)	UPLC IMS QTOF	[59]
Can coatings	Diisobutyl phthalate (DIBP), Degradation products formed from antioxidants (1,3-di-tert-butylbenzene and 2,4-di-tert-butylphenol degradation products from antioxidants Irgafos 168 or Irganox 1076, 2,6-di-tert-butyl-1,4-benzoquinone degradation products from antioxidants Irgafos 168 and Irganox 1010, 7,9-di-tert-butyl-1-oxaspiro(4,5)deca-6,9-diene-2,8-dione, a degradation product of Irganox 1010.	GC-MS and LC-MS/MS	[78]
Low-density polyethylene-LDPE and polyamide-PA added of NBBS, α-MSD, Irganox 1081, Irganox 1222, Santonox; LDPE 2/PA 6 2: Nonox A, Neozon D, Antioxidant 2246, Tinuvin P, TOTM	Degradation products of TOTM, including DEHP, isophthalate bis(2-ethylhexyl)benzene-1,3-dicarboxylate (DOIP); decomposition product of NBBS was *N*-ethyl-*N*-methylbenzenesulfonamide; The formation of the cyclic saturated isomer (1,1,3-trimethyl-3-phenyl-2H-indene) is triggered by the thermal impact, and so is the rearrangement of the carbon double bond to form isomer 2,4-diphenyl-4-methyl-2(E)-pentene). Decomposition product 2,3-dimethyl-3-phenylbutan-2-yl)benzene is formed by combining two cumyl radicals during pyrolysis of the pure additive and pyrolysis of LDPE entailing α-MSD. The degradation product of Neozon D and Nonox A identified in oxidative pyrolysis of the pure analyte was 10-methyl-benz[a]acridine; degradation products of Antioxidant 2246, Santonox, Irganox 1222/1081 and Tinuvin P: o-cresol, m-cresol or p-cresol, 2-tert-butyl-4-methylphenol and 2-tert-butyl-4,6-dimethylphenol, 3,5-di-tert-butyl-4-hydroxybenzaldehyde	Pyr-GC–MS and GC-EI-MS/MS	[104]
Recycled pellets obtained from post-consumer low-density polyethylene (PC-LDPE) and high-density polyethylene (PC-HPDE)	Polymer degradation products: octanal and nonanal (aldehydes); 3-decanone, 2-undecanone, 2,2,4,4,6,8,8-heptamethylnonanone and 3-dodecanone (ketones); hexane (others); Additives degradation products: 3,5-di-tert-butyl-4-hydroxybenzaldehyde (aldehyde); 2,6-di-tert-butyl-1,4-benzoquinone and 3,5-di-tert-butyl-4-hydroxyacetophenone (ketones); methyl tetradecanoate, ethyl tetradecanoate and ethyl palmitate(esters); 7,9-di-tert-butyl-1-oxaspiro (4,5)deca-6,9-diene-2,8-dione (others); Contaminants from external sources: methyl lactate, hexyl acetate, and dimethyl butanedioate, α-methylionone, 3-(4-Isopropylphenyl)-2-methylpropionaldehyde, α-amylcinnamaldehyde, (phenylmethylene)octanal and dipropylene glycol amog others (cosmetic ingredients); alkylbenzenes (breakdown products produced by the degradation of alkylbenzene sulfonates); contamination related to food: the lactones, 5-methylfurfural, furfural and methyl hexanoate (can derive from food flavors as well as from cosmetics ingredients); furfuryl alcohol, methyl pyruvate and 2-acetyl pyridine (food flavors), methyl-2-ethylhexanoate, acetic acid, propanoic acid, pyridine and dimethyl trisulfide (rotten food products), 2,6-diisopropylnaphthalene (paper labels).	GC/MS and HS-SPME-GC/MS	[79]

GC × GC−ToF MS: two-dimensional gas chromatography-time-of-flight mass spectrometry; GC-FID–MS: gas chromatography-mass spectrometry and flame ionization detector; GC-MS: gas chromatography-mass spectrometry; HPLC-DAD: high-performance liquid chromatographic method with diode-array detection; HS-SPME-GC-MS: a combination of headspace solid-phase microextraction (HS-SPME) and gas chromatography-mass spectrometry; LC-MS/MS: Liquid chromatography-tandem mass spectrometry Pyr-GC–MS: Pyrolysis gas chromatography-mass spectrometry; UHPLC–ESI Q-orbitrap: ultra-high-performance liquid chromatography-electrospray ionization quadrupole Orbitrap mass spectrometry; UHPLC–Q-TOF/MSE: ultra-performance liquid chromatography coupled with a hybrid quadrupole time-of-flight mass spectrometry; UPLC IMS QTOF: ultra-performance liquid chromatography-ion mobility separation-quadruple time-of-flight; UPLC-ESI-MS/QTOF: ultra-performance liquid chromatography-electrospray ionization mass spectrometer quadrupole time of flight.

**Table 5:** Studies published in the literature on impurities (NIAS) Identification in different plastic food packaging.

Food Contact Material	NIAS	Method/Technique	Reference
Active packaging: Polypropylene (PP); PP + green tea; PP/poly (ethylene-co-vinyl alcohol) EVOH; PP/EVOH + oregano; PP/EVOH + citral; EVOH; Polyethylene terephthalate-PET/EVOH + citral; PET/EVOH + cinnamon; PP/EVOH/PP; PP/EVOH + oregano	Degradation of active compounds; impurities from the raw materials; additives used in the manufacture of the active polymer (citral thermal reaction products; oxidation product of citral; decomposition product of adipates used as plasticizers; impurity/reaction product/breakdown product for the additives used in the manufacture of PE materials; xanthenone derivates)	UPLC-QTOF-MS	[86]
Polyethylene terephthalate-PET film with an acrylic resin	Ethyl lauroyl arginate (LAE) impurities: N2-Dodecanoyl-l-arginine (LAS)	UPLC–MS(QTOF)	[87]
Polypropylene (PP)	Degradation products derived from phenolic antioxidants, impurity/reaction product/breakdown product of the additives, Family 1: Family formed with the reference structure; was formed by the compounds that had a similar structure constituted by a group 3,5-di-tert-butyl-4-hydroxyphenyl; Family 2: With glycerol molecule (glyceryl monostearate, glyceryl palmitate and glyceryl dihexadecanoate, an ester of an acid chain bonded to a glycerol molecule); Family 3: Dihydroxy alquilamines (amine bonded to two ethanol molecules and also an alkyl hydrocarbon chain); Family 4: ceramide and dihydroceramide (a family of waxy lipid molecules which are composed of sphingosine (an 18 carbon amino alcohol with an unsaturated hydrocarbon chain) and a fatty acid); Family 5: amides bonded by ethylene (degradation products from a lubricant losing C_2_H_4_); Other compounds (amides come from the impurities or degradation products from erucamide and oleamide widely used as slip agents)	UPLC-MS-QTOF	[71]
Polyester-polyurethane lacquers	Impurities or degradation products of IPDI trimer IPDI and DPMDI, two cyclic oligoesters, 2EG + 2TPA and 2NPG + 2oPA	GC-(EI)qMS, GC-(EI)Orbitrap, GC-(APCI)TOFHRMS and GC(×GC)-(EI)TOFLRMS	[96]
Plastic baby bibs (polyethylene vinyl acetate-PEVA, polyamide-PA and polyethylene-PE)	Azocine, octahydro-1-nitroso-(Possible NIAS from printing ink); 1,6-Dioxacyclododecane-7,12-dione (NIAS from polyurethane adhesive); 1-Propene-1,2,3-tricarboxylic acid, tributyl ester (Tributyl aconitate)	GC-MS	[80]

GC-(APCI)TOFHRMS: gas chromatography atmospheric-pressure chemical ionization time-of-flight high-resolution mass spectrometry; GC-(EI)Orbitrap: gas chromatography/electron ionization Orbitrap; GC-(EI)qMS: gas chromatography/electron ionization-quadrupole mass spectrometry; GC × GC−ToF MS: two-dimensional gas chromatography-time-of-flight mass spectrometry; GC-MS: gas chromatography-mass spectrometry; UPLC-QTOF-MS; UPLC–MS(QTOF): ultra-performance liquid chromatography–quadrupole time of flight mass spectrometry.

**Table 6:** Studies published in the literature on contaminants (NIAS) Identification in different plastic food packaging.

Food Contact Material	NIAS	Method/Technique	Reference
Rigid thermoformed containers and films made with Recycled polyethylene terephthalate-RPET	Chromium, nickel	ICP-AES	[31]
Recycled pellets obtained from post-consumer low-density polyethylene (PC-LDPE) and high-density polyethylene (PC-HPDE)	Contaminants from external sources: methyl lactate, hexyl acetate, and dimethyl butanedioate, α-methylionone, 3-(4-Isopropylphenyl)-2-methylpropionaldehyde, α-amylcinnamaldehyde, (phenylmethylene)octanal and dipropylene glycol amog others (cosmetic ingredients); alkylbenzenes (breakdown products produced by the degradation of alkylbenzene sulfonates); contamination related to food: the lactones, 5-methylfurfural, furfural and methyl hexanoate (can derive from food flavors as well as from cosmetics ingredients); furfuryl alcohol, methyl pyruvate and 2-acetyl pyridine (food flavors), methyl-2-ethylhexanoate, acetic acid, propanoic acid, pyridine and dimethyl trisulfide (rotten food products), 2,6-diisopropylnaphthalene (paper labels).	GC/MS and HS-SPME-GC/MS	[79]
Low-Density Polyethylene (LDPE) films	Calcite (CaCO_3_), calcium sulphate (CaSO_4_), polystyrene (PS) and titanium dioxide (TiO2), Ca and Ti	Raman spectroscopy and ICP-MS	[99]

GC-MS: gas chromatography-mass spectrometry; ICP-AES: inductively coupled plasma atomic emission spectrometry; ICP-MS: inductively coupled plasma mass spectrometry; HS-SPME-GC-MS: a combination of headspace solid-phase microextraction (HS-SPME) and gas chromatography-mass spectrometry.

**Table 7:** Food simulants recommended by the (EU) n° 10/2011 and examples of applications.

Food Simulant	Recommendation to Use	Examples of Food
Ethanol 10% (*v*/*v*) (A)	Hydrophilic character and can extract hydrophilic Substances	Fresh vegetables, peeled or cut
Acetic acid 3% (*w*/*v*) (B)	Hydrophilic character and can extract hydrophilic substances; food with pH < 4.5	Clear drinks: Water, ciders, clear fruit or vegetable juices of normal strength or concentrated, fruit nectars, lemonades, syrups, bitters, infusions, coffee, tea, beers, soft drinks, energy drinks and the like, flavored water, liquid coffee extract
Ethanol 20% (*v*/*v*) (C)	Hydrophilic character and can extract hydrophilic substances; alcoholic foods with an alcohol content of up to 20% and those foods which contain a relevant amount of organic ingredients that render the food more lipophilic	Ice-creams
Ethanol 50% (*v*/*v*) (D1)	Lipophilic character and can extract lipophilic substances; for alcoholic foods with an alcohol content of above 20% and oil in water Emulsions	Milk and milk-based drinks whole, partly dried and skimmed or partly skimmed
Vegetable oil (D2)	Lipophilic character and can extract lipophilic substances; foods which contain free fats at the surface	Animals and vegetable fats and oils, whether natural or treated (including cocoa butter, lard, resolidified butter)
Poly (2,6-diphenyl-p-phenylene oxide), particle size 60–80 mesh, pore size 200 nm (E)	For testing specific migration into dry foods	Cereal flour and meal

## References

[1] Nerin C., Alfaro P., Aznar M., Domeño C. (2013). The challenge of identifying non-intentionally added substances from food packaging materials: A review. Anal. Chim. Acta.

[2] Leeman W., Krul L. (2015). Non-intentionally added substances in food contact materials: How to ensure consumer safety. Curr. Opin. Food Sci..

[3] Bignardi C., Cavazza A., Laganà C., Salvadeo P., Corradini C. (2017). Release of non-intentionally added substances (NIAS) from food contact polycarbonate: Effect of ageing. Food Control..

[4] Martínez-Bueno M.J., Gómez Ramos M.J., Bauer A., Fernández-Alba A.R. (2019). An overview of non-targeted screening strategies based on high resolution accurate mass spectrometry for the identification of migrants coming from plastic food packaging materials. TrAC Trends Anal. Chem..

[5] Wrona M., Nerín C. (2020). Analytical Approaches for Analysis of Safety of Modern Food Packaging: A Review. Molecules.

[6] Kim H.S., Lee Y.J., Koo Y.J., Pack E.C., Lim K.M., Choi D.W. (2021). Migration of monomers, plastic additives, and non-intentionally added substances from food utensils made of melamine–formaldehyde resin following ultraviolet sterilization. Food Control..

[7] Koster S., Bani-Estivals M.H., Bonuomo M., Bradley E., Chagnon M.C., García M.L., Godts F., Gude T., Helling R., Paseiro-Losada P. (2015). Guidance on Best Practices on the Risk Assessment of Non-Intentionally Added Substances (Nias) in Food Contact Materials and Articles.

[8] EFSA, WHO (2016). European Food Safety Authority and World Health Organization Review of the Threshold of Toxicological Concern (TTC) approach and development of new TTC decision tree. EFSA Support. Publ..

[9] Peters R.J.B., Groeneveld I., Sanchez P.L., Gebbink W., Gersen A., de Nijs M., van Leeuwen S.P.J. (2019). Review of analytical approaches for the identification of non-intentionally added substances in paper and board food contact materials. Trends Food Sci. Technol..

[10] Wang Y., Gao X., Liu B., Lin Q., Xia Y. (2020). Identification of chemicals in a polyvinyl chloride/polyethylene multilayer film by ultra-high-performance liquid chromatography/quadrupole time-of-flight mass spectrometry and their migration into solution. J. Chromatogr. A.

[11] U.S. FDA CFR—Code of Federal Regulations Title 21. https://www.accessdata.fda.gov/scripts/cdrh/cfdocs/cfcfr/CFRSearch.cfm?fr=174.5.

[12] Karmaus A.L., Osborn R., Krishan M. (2018). Scientific advances and challenges in safety evaluation of food packaging materials: Workshop proceedings. Regul. Toxicol. Pharmacol..

[13] Souza V.G.L., Fernando A.L. (2016). Nanoparticles in food packaging: Biodegradability and potential migration to food—A review. Food Packag. Shelf Life.

[14] Yates J., Deeney M., Rolker H.B., White H., Kalamatianou S., Kadiyala S. (2021). A systematic scoping review of environmental, food security and health impacts of food system plastics. Nat. Food.

[15] Ong H.-T., Samsudin H., Soto-Valdez H. (2020). Migration of endocrine-disrupting chemicals into food from plastic packaging materials: An overview of chemical risk assessment, techniques to monitor migration, and international regulations. Crit. Rev. Food Sci. Nutr..

[16] Rodrigues M.O., Abrantes N., Gonçalves F.J.M., Nogueira H., Marques J.C., Gonçalves A.M.M. (2019). Impacts of plastic products used in daily life on the environment and human health: What is known?. Environ. Toxicol. Pharmacol..

[17] Galotto M.J., Torres A., Guarda A., Moraga N., Romero J. (2011). Experimental and theoretical study of LDPE versus different concentrations of Irganox 1076 and different thickness. Food Res. Int..

[18] Hahladakis J.N., Velis C.A., Weber R., Iacovidou E., Purnell P. (2018). An overview of chemical additives present in plastics: Migration, release, fate and environmental impact during their use, disposal and recycling. J. Hazard. Mater..

[19] Groh K.J., Backhaus T., Carney-Almroth B., Geueke B., Inostroza P.A., Lennquist A., Leslie H.A., Maffini M., Slunge D., Trasande L. (2019). Overview of known plastic packaging-associated chemicals and their hazards. Sci. Total Environ..

[20] Geueke B., Wagner C.C., Muncke J. (2014). Food contact substances and chemicals of concern: A comparison of inventories. Food Addit. Contam. Part A Chem. Anal. Control. Expo. Risk Assess..

[21] European Commission (2011). Commission Regulation (EU) No 10/2011 of 14 January 2011 on Plastic Materials and Articles Intended to Come Into contact With Food. Off. J. Eur. Union..

[22] Boucher J. (2019). Mercosur Positive List for FCM Plastic Additives. https://www.foodpackagingforum.org/news/mercosur-positive-list-for-fcm-plastic-additives.

[23] Muncke J., Backhaus T., Geueke B., Maffini M.V., Martin O.V., Myers J.P., Soto A.M., Trasande L., Trier X., Scheringer M. (2017). Scientific Challenges in the Risk Assessment of Food Contact Materials. Environ. Health Perspect..

[24] Bhunia K., Sablani S.S., Tang J., Rasco B. (2013). Migration of Chemical Compounds from Packaging Polymers during Microwave, Conventional Heat Treatment, and Storage. Compr. Rev. Food Sci. Food Saf..

[25] Ubeda S., Aznar M., Rosenmai A.K., Vinggaard A.M., Nerín C. (2020). Migration studies and toxicity evaluation of cyclic polyesters oligomers from food packaging adhesives. Food Chem..

[26] Mistura L., Sette S., O’Mahony C., Engel K.-H., Mehegan J., Leclercq C. (2013). Modelling framework for assessment of dietary exposure to added flavouring substances within the FACET (Flavours, Additives, and Food Contact Material Exposure Task) project. Food Chem. Toxicol..

[27] Grob K., Biedermann M., Scherbaum E., Roth M., Rieger K. (2006). Food contamination with organic materials in perspective: Packaging materials as the largest and least controlled source? A view focusing on the European situation. Crit. Rev. Food Sci. Nutr..

[28] Hoppe M., Fornari R., de Voogt P., Franz R. (2017). Migration of oligomers from PET: Determination of diffusion coefficients and comparison of experimental versus modelled migration. Food Addit. Contam. Part A.

[29] Eckardt M., Hetzel L., Brenz F., Simat T.J. (2020). Release and migration of cyclic polyester oligomers from bisphenol A non-intent polyester–phenol-coatings into food simulants and infant food—A comprehensive study. Food Addit. Contam. Part A.

[30] Fasano E., Bono-Blay F., Cirillo T., Montuori P., Lacorte S. (2012). Migration of phthalates, alkylphenols, bisphenol A and di(2-ethylhexyl)adipate from food packaging. Food Control..

[31] Whitt M., Brown W., Danes J.E., Vorst K.L. (2016). Migration of heavy metals from recycled polyethylene terephthalate during storage and microwave heating. J. Plast. Film Sheeting.

[32] Clemente I., Aznar M., Nerín C., Bosetti O. (2016). Migration from printing inks in multilayer food packaging materials by GC-MS analysis and pattern recognition with chemometrics. Food Addit. Contam. Part A.

[33] Isella F., Canellas E., Bosetti O., Nerin C. (2013). Migration of non intentionally added substances from adhesives by UPLC-Q-TOF/MS and the role of EVOH to avoid migration in multilayer packaging materials. J. Mass Spectrom..

[34] Song X.-C., Wrona M., Nerin C., Lin Q.-B., Zhong H.-N. (2019). Volatile non-intentionally added substances (NIAS) identified in recycled expanded polystyrene containers and their migration into food simulants. Food Packag. Shelf Life.

[35] Helmroth I.E., Bekhuis H.A.M., Linssen J.P.H., Dekker M. (2002). Direct measurement of additive migration from low-density polyethylene as a function of space and time. J. Appl. Polym. Sci..

[36] Lee D.S., Yam K.L., Piergiovanni L. (2008). Food Packaging Science and Technology.

[37] Alberto Lopes J., Tsochatzis E.D., Karasek L., Hoekstra E.J., Emons H. (2021). Analysis of PBT and PET cyclic oligomers in extracts of coffee capsules and food simulants by a HPLC-UV/FLD method. Food Chem..

[38] Committee E.S., More S.J., Bampidis V., Benford D., Bragard C., Halldorsson T.I., Hernández-Jerez A.F., Hougaard Bennekou S., Koutsoumanis K.P., Machera K. (2019). Guidance on the use of the Threshold of Toxicological Concern approach in food safety assessment. EFSA J..

[39] Eckardt M., Kubicova M., Simat T.J. (2018). Universal response quantification approach using a Corona Charged Aerosol Detector (CAD)—Application on linear and cyclic oligomers extractable from polycondensate plastics polyesters, polyamides and polyarylsulfones. J. Chromatogr. A.

[40] Nelson C.P., Patton G.W., Arvidson K., Lee H., Twaroski M.L. (2011). Assessing the toxicity of polymeric food-contact substances. Food Chem. Toxicol..

[41] Muncke J., Myers J.P., Scheringer M., Porta M. (2014). Food packaging and migration of food contact materials: Will epidemiologists rise to the neotoxic challenge?. J. Epidemiol. Community Health.

[42] Arvanitoyannis I.S., Kotsanopoulos K.V. (2014). Migration Phenomenon in Food Packaging. Food–Package Interactions, Mechanisms, Types of Migrants, Testing and Relative Legislation—A Review. Food Bioprocess Technol..

[43] Fang X., Vitrac O. (2017). Predicting diffusion coefficients of chemicals in and through packaging materials. Crit. Rev. Food Sci. Nutr..

[44] Xue M., Chai X.-S., Li X., Chen R. (2019). Migration of organic contaminants into dry powdered food in paper packaging materials and the influencing factors. J. Food Eng..

[45] Nerín C., Aznar M., Carrizo D. (2016). Food contamination during food process. Trends Food Sci. Technol..

[46] Tehrany E.A.T., Desobry S. (2004). Partition coefficients in food/packaging systems: A review. Food Addit. Contam..

[47] Arvanitoyannis I.S., Bosnea L. (2004). Migration of substances from food packaging materials to foods. Crit. Rev. Food Sci. Nutr..

[48] Begley T.H., Biles J.E., Cunningham C., Piringer O. (2004). Migration of a UV stabilizer from polyethylene terephthalate (PET) into food simulants. Food Addit. Contam..

[49] Ferrara G., Bertoldo M., Scoponi M., Ciardelli F. (2001). Diffusion coefficient and activation energy of Irganox 1010 in poly(propylene-co-ethylene) copolymers. Polym. Degrad. Stab..

[50] Chung D., Papadakis S.E., Yam K.L. (2002). Simple models for assessing migration from food-packaging films. Food Addit. Contam..

[51] O’Brien A., Cooper L. (2001). Polymer additive migration to foods—A direct comparison of experimental data and values calculated from migration models for polypropylene. Food Addit. Contam..

[52] Brandsch J., Mercea P., Rüter M., Tosa V., Piringer O. (2002). Migration modelling as a tool for quality assurance of food packaging. Food Addit. Contam..

[53] Begley T., Castle L., Feigenbaum A., Franz R., Hinrichs K., Lickly T., Mercea P., Milana M., O’Brien A., Rebre S. (2005). Evaluation of migration models that might be used in support of regulations for food-contact plastics. Food Addit. Contam..

[54] Bott J., Störmer A., Franz R. (2014). A model study into the migration potential of nanoparticles from plastics nanocomposites for food contact. Food Packag. Shelf Life.

[55] Gavriil G., Kanavouras A., Coutelieris F.A. (2018). Food-packaging migration models: A critical discussion. Crit. Rev. Food Sci. Nutr..

[56] Oldring P.K.T., O’Mahony C., Dixon J., Vints M., Mehegan J., Dequatre C., Castle L. (2014). Development of a new modelling tool (FACET) to assess exposure to chemical migrants from food packaging. Food Addit. Contam. Part A.

[57] Katiyar V. (2020). Sustainable Polymers for Food Packaging.

[58] Genualdi S., Nyman P., Begley T. (2014). Updated evaluation of the migration of styrene monomer and oligomers from polystyrene food contact materials to foods and food simulants. Food Addit. Contam. Part A.

[59] Vera P., Canellas E., Barknowitz G., Goshawk J., Nerín C. (2019). Ion-Mobility Quadrupole Time-of-Flight Mass Spectrometry: A Novel Technique Applied to Migration of Nonintentionally Added Substances from Polyethylene Films Intended for Use as Food Packaging. Anal. Chem..

[60] Paseiro-Cerrato R., MacMahon S., Ridge C.D., Noonan G.O., Begley T.H. (2016). Identification of unknown compounds from polyester cans coatings that may potentially migrate into food or food simulants. J. Chromatogr. A.

[61] Groh K.J., Geueke B., Martin O., Maffini M., Muncke J. (2021). Overview of intentionally used food contact chemicals and their hazards. Environ. Int..

[62] Grob K. (2014). Work plans to get out of the deadlock for the safety assurance of migration from food contact materials? A proposal. Food Control..

[63] Driffield M., Garcia-Lopez M., Christy J., Lloyd A.S., Tarbin J.A., Hough P., Bradley E.L., Oldring P.K.T. (2018). The determination of monomers and oligomers from polyester-based can coatings into foodstuffs over extended storage periods. Food Addit. Contam. Part A.

[64] Pietropaolo E., Albenga R., Gosetti F., Toson V., Koster S., Marin-Kuan M., Veyrand J., Patin A., Schilter B., Pistone A. (2018). Synthesis, identification and quantification of oligomers from polyester coatings for metal packaging. J. Chromatogr. A.

[65] Ubeda S., Aznar M., Nerín C. (2018). Determination of oligomers in virgin and recycled polyethylene terephthalate (PET) samples by UPLC-MS-QTOF. Anal. Bioanal. Chem..

[66] Bauer A., Jesús F., Gómez Ramos M.J., Lozano A., Fernández-Alba A.R. (2019). Identification of unexpected chemical contaminants in baby food coming from plastic packaging migration by high resolution accurate mass spectrometry. Food Chem..

[67] Felix J.S., Isella F., Bosetti O., Nerin C. (2012). Analytical tools for identification of non-intentionally added substances (NIAS) coming from polyurethane adhesives in multilayer packaging materials and their migration into food simulants. Anal. Bioanal. Chem..

[68] Canellas E., Vera P., Nerín C. (2019). Ion mobility quadrupole time-of-flight mass spectrometry for the identification of non-intentionally added substances in UV varnishes applied on food contact materials. A safety by design study. Talanta.

[69] Pezo D., Fedeli M., Bosetti O., Nerín C. (2012). Aromatic amines from polyurethane adhesives in food packaging: The challenge of identification and pattern recognition using Quadrupole-Time of Flight-Mass SpectrometryE. Anal. Chim. Acta.

[70] Yan Y., Hu C.-Y., Wang Z.-W., Jiang Z.-W. (2018). Degradation of Irgafos 168 and migration of its degradation products from PP-R composite films. Packag. Technol. Sci..

[71] Vera P., Canellas E., Nerín C. (2018). Identification of non volatile migrant compounds and NIAS in polypropylene films used as food packaging characterized by UPLC-MS/QTOF. Talanta.

[72] Vera P., Canellas E., Nerín C. (2013). Identification of non-volatile compounds and their migration from hot melt adhesives used in food packaging materials characterized by ultra-performance liquid chromatography coupled to quadrupole time-of-flight mass spectrometry. Anal. Bioanal. Chem..

[73] Kassouf A., Maalouly J., Chebib H., Rutledge D.N., Ducruet V. (2013). Chemometric tools to highlight non-intentionally added substances (NIAS) in polyethylene terephthalate (PET). Talanta.

[74] Riquet A.M., Breysse C., Dahbi L., Loriot C., Severin I., Chagnon M.C. (2016). The consequences of physical post-treatments (microwave and electron-beam) on food/packaging interactions: A physicochemical and toxicological approach. Food Chem..

[75] Garcia Ibarra V., de Quiros A.R., Paseiro Losada P., Sendon R. (2019). Non-target analysis of intentionally and non intentionally added substances from plastic packaging materials and their migration into food simulants. Food Packag. Shelf Life.

[76] Garcia Ibarra V., de Quiros A.R., Paseiro Losada P., Sendon R. (2018). Identification of intentionally and non-intentionally added substances in plastic packaging materials and their migration into food products. Anal. Bioanal. Chem..

[77] Carrero-Carralero C., Escobar-Arnanz J., Ros M., Jiménez-Falcao S., Sanz M.L., Ramos L. (2019). An untargeted evaluation of the volatile and semi-volatile compounds migrating into food simulants from polypropylene food containers by comprehensive two-dimensional gas chromatography-time-of-flight mass spectrometry. Talanta.

[78] Lestido Cardama A., Sendón R., Bustos J., Santillana M.I., Paseiro Losada P., Rodríguez Bernaldo de Quirós A. (2019). GC-MS Screening for the Identification of Potential Migrants Present in Polymeric Coatings of Food Cans. Polymers.

[79] Horodytska O., Cabanes A., Fullana A. (2020). Non-intentionally added substances (NIAS) in recycled plastics. Chemosphere.

[80] Rajbux C., Pereira J., do Céu Selbourne M., Costa-Pinto A.R., Poças F. (2020). Assessment of baby Bibs. GC-MS screening, migration into saliva and insight of toxicity with QSAR tools. Food Control..

[81] Tsochatzis E.D., Gika H., Theodoridis G. (2020). Development and validation of a fast gas chromatography mass spectrometry method for the quantification of selected non-intentionally added substances and polystyrene/polyurethane oligomers in liquid food simulants. Anal. Chim. Acta.

[82] Carrizo D., Maccagnan A., Felix J.S., Nerin C., Bosetti O. (2015). The Barrier Effect of EVOH versus 1,4,7-Triaxocyclotridecane-8,13-Dione, a Non-intentionally Added Compound from Polyurethane Adhesives in Multilayer Food Packaging. Packag. Technol. Sci..

[83] Vaclavikova M., Paseiro-Cerrato R., Vaclavik L., Noonan G.O., De Vries J., Begley T.H. (2016). Target and non-target analysis of migrants from PVC-coated cans using UHPLC-Q-Orbitrap MS: Evaluation of long-term migration testing. Food Addit. Contam. Part A.

[84] Martinez-Bueno M.J., Hernando M.D., Ucles S., Rajski L., Cimmino S., Fernandez-Alba A.R. (2017). Identification of non-intentionally added substances in food packaging nano films by gas and liquid chromatography coupled to orbitrap mass spectrometry. Talanta.

[85] Úbeda S., Aznar M., Vera P., Nerín C., Henríquez L., Taborda L., Restrepo C. (2017). Overall and specific migration from multilayer high barrier food contact materials—Kinetic study of cyclic polyester oligomers migration. Food Addit. Contam. Part A.

[86] Aznar M., Rodriguez-Lafuente A., Alfaro P., Nerin C. (2012). UPLC-Q-TOF-MS analysis of non-volatile migrants from new active packaging materials. Anal. Bioanal. Chem..

[87] Aznar M., Gómez-Estaca J., Vélez D., Devesa V., Nerín C. (2013). Migrants determination and bioaccessibility study of ethyl lauroyl arginate (LAE) from a LAE based antimicrobial food packaging material. Food Chem. Toxicol..

[88] Aznar M., Domeno C., Nerin C., Bosetti O. (2015). Set-off of non volatile compounds from printing inks in food packaging materials and the role of lacquers to avoid migration. Dye Pigment.

[89] Helling R., Seifried P., Fritzsche D., Simat T.J. (2012). Characterisation and migration properties of silicone materials during typical long-term commercial and household use applications: A combined case study. Food Addit. Contam. Part A.

[90] Yan J.W., Hu C., Tong L.H., Lei Z.X., Lin Q.-B. (2020). Migration test and safety assessment of polyurethane adhesives used for food-contact laminated films. Food Packag. Shelf Life.

[91] Panseri S., Chiesa L.M., Zecconi A., Soncini G., De Noni I. (2014). Determination of Volatile Organic Compounds (VOCs) from wrapping films and wrapped PDO Italian cheeses by using HS-SPME and GC/MS. Molecules.

[92] Aznar M., Alfaro P., Nerin C., Kabir A., Furton K.G. (2016). Fabric phase sorptive extraction: An innovative sample preparation approach applied to the analysis of specific migration from food packaging. Anal. Chim. Acta.

[93] Kuki Á., Nagy L., Nagy T., Zsuga M., Kéki S. (2017). Screening of additives and other chemicals in polyurethanes by direct analysis in real time mass spectrometry (DART-MS). Anal. Bioanal. Chem..

[94] Bignardi C., Cavazza A., Corradini C., Salvadeo P. (2014). Targeted and untargeted data-dependent experiments for characterization of polycarbonate food-contact plastics by ultra high performance chromatography coupled to quadrupole orbitrap tandem mass spectrometry. J. Chromatogr. A.

[95] Galmán Graíño S., Sendón R., López Hernández J., Rodríguez-Bernaldo de Quirós A., Graíño S.G., Sendón R., Hernández J.L., de Quirós A.R.-B. (2018). GC-MS screening analysis for the identification of potential migrants in plastic and paper-based candy wrappers. Polymers.

[96] Omer E., Bichon E., Hutinet S., Royer A.-L., Monteau F., Germon H., Hill P., Remaud G., Dervilly-Pinel G., Cariou R. (2019). Toward the characterisation of non-intentionally added substances migrating from polyester-polyurethane lacquers by comprehensive gas chromatography-mass spectrometry technologies. J. Chromatogr. A.

[97] Brenz F., Linke S., Simat T. (2018). Linear and cyclic oligomers in polybutylene terephthalate for food contact materials. Food Addit. Contam. Part A.

[98] Gómez Ramos M.J., Lozano A., Fernández-Alba A.R. (2019). High-resolution mass spectrometry with data independent acquisition for the comprehensive non-targeted analysis of migrating chemicals coming from multilayer plastic packaging materials used for fruit purée and juice. Talanta.

[99] Portesi C., Visentin D., Durbiano F., Abete M.C., Rizzi M., Maurino V., Rossi A.M. (2019). Development of a rapid micro-Raman spectroscopy approach for detection of NIAS in LDPE pellets and extruded films for food packaging applications. Polym. Test..

[100] Canellas E., Vera P., Nerín C. (2017). Migration assessment and the “threshold of toxicological concern” applied to the safe design of an acrylic adhesive for food-contact laminates. Food Addit. Contam. Part A.

[101] Habchi B., Kassouf A., Padellec Y., Rathahao-Paris E., Alves S., Rutledge D.N., Maalouly J., Ducruet V. (2018). An untargeted evaluation of food contact materials by flow injection analysis-mass spectrometry (FIA-MS) combined with independent components analysis (ICA). Anal. Chim. Acta.

[102] Salafranca J., Clemente I., Isella F., Nerín C., Bosetti O. (2015). Influence of oxygen and long term storage on the profile of volatile compounds released from polymeric multilayer food contact materials sterilized by gamma irradiation. Anal. Chim. Acta.

[103] Paseiro-Cerrato R., DeJager L., Begley T.H. (2019). Assessment of the Impact of Accelerated Migration Testing for Coated Food Cans Using Food Simulants. Molecules.

[104] Bartsch N., Girard M., Wilde A., Bruhn T., Kappenstein O., Vieth B., Hutzler C., Luch A. (2019). Thermal Stability of Polymer Additives: Comparison of Decomposition Models Including Oxidative Pyrolysis. J. Vinyl. Addit. Technol..

[105] Hoppe M., de Voogt P., Franz R. (2016). Identification and quantification of oligomers as potential migrants in plastics food contact materials with a focus in polycondensates—A review. Trends Food Sci. Technol..

[106] EFSA Panel on Food Contact Materials, Enzymes, Flavourings and Processing Aids (CEF) (2014). Scientific Opinion on the safety assessment of the substance, furan-2, 5-dicarboxylic acid, CAS No 3238-40-2, for use in food contact materials. EFSA J..

[107] Gelbke H.-P., Banton M., Block C., Dawkins G., Leibold E., Pemberton M., Sakoda A., Yasukawa A. (2018). Oligomers of styrene are not endocrine disruptors. Crit. Rev. Toxicol..

[108] Paseiro-Cerrato R., Noonan G.O., Begley T.H. (2016). Evaluation of Long-Term Migration Testing from Can Coatings into Food Simulants: Polyester Coatings. J. Agric. Food Chem..

[109] Campanella G., Ghaani M., Quetti G., Farris S. (2015). On the origin of primary aromatic amines in food packaging materials. Trends Food Sci. Technol..

[110] Wrona M., Nerin C. (2019). CHAPTER 7 Risk Assessment of Plastic Packaging for Food Applications. Food Contact Materials Analysis: Mass Spectrometry Techniques.

[111] McCombie G., Hötzer K., Daniel J., Biedermann M., Eicher A., Grob K. (2016). Compliance work for polyolefins in food contact: Results of an official control campaign. Food Control..

[112] Canellas E., Aznar M., Nerin C., Mercea P. (2010). Partition and diffusion of volatile compounds from acrylic adhesives used for food packaging multilayers manufacturing. J. Mater. Chem..

[113] Biedermann M., Grob K. (2006). Polyadipates used as plasticizers in food contact: Fraction below 1000 Da determined by size exclusion chromatography with evaporative light scattering detection and segmental response linearization or UV detection. J. Sep. Sci..

[114] Groh K.J., Muncke J. (2017). In Vitro Toxicity Testing of Food Contact Materials: State-of-the-Art and Future Challenges. Compr. Rev. Food Sci. Food Saf..

[115] Sanchis Y., Yusà V., Coscollà C. (2017). Analytical strategies for organic food packaging contaminants. J. Chromatogr. A.

[116] Yusà V., López A., Dualde P., Pardo O., Fochi I., Pineda A., Coscolla C. (2020). Analysis of unknowns in recycled LDPE plastic by LC-Orbitrap Tribrid HRMS using MS3 with an intelligent data acquisition mode. Microchem. J..

[117] Aznar M., Alfaro P., Nerín C., Jones E., Riches E. (2016). Progress in mass spectrometry for the analysis of set-off phenomena in plastic food packaging materials. J. Chromatogr. A.

[118] Hakkarainen M., Karlsson S. (2006). Gas Chromatography in Analysis of Polymers and Rubbers. Encyclopedia of Analytical Chemistry.

[119] Peñalver R., Arroyo-Manzanares N., Campillo N., Viñas P. (2021). Targeted and untargeted gas chromatography-mass spectrometry analysis of honey samples for determination of migrants from plastic packages. Food Chem..

[120] Wang H., Yuan J. (2016). Identification and quantification of unknown antioxidants in plastic materials by ultrasonic extraction and ultra-performance liquid chromatography coupled with quadrupole time-of-flight mass spectrometry. Eur. J. Mass Spectrom..

[121] Canellas E., Vera P., Domeno C., Alfaro A.P., Nerin C. (2012). Atmospheric pressure gas chromatography coupled to quadrupole-time of flight mass spectrometry as a powerful tool for identification of non intentionally added substances in acrylic adhesives used in food packaging materials. J. Chromatogr. A.

[122] Omer E., Cariou R., Remaud G., Guitton Y., Germon H., Hill P., Dervilly-Pinel G., Le Bizec B. (2018). Elucidation of non-intentionally added substances migrating from polyester-polyurethane lacquers using automated LC-HRMS data processing. Anal. Bioanal. Chem..

[123] Canellas E., Vera P., Nerin C. (2015). UPLC-ESI-Q-TOF-MSE and GC-MS identification and quantification of non-intentionally added substances coming from biodegradable food packaging. Anal. Bioanal. Chem..

[124] Špánik I., Machyňáková A. (2018). Recent applications of gas chromatography with high-resolution mass spectrometry. J. Sep. Sci..

[125] Hernández F., Portolés T., Pitarch E., López F.J. (2011). Gas chromatography coupled to high-resolution time-of-flight mass spectrometry to analyze trace-level organic compounds in the environment, food safety and toxicology. TrAC Trends Anal. Chem..

[126] Hoppe M., De Voogt P., Franz R. (2018). Oligomers in polyethylene furanoate—Identification and quantification approach via LC-UV LC-MS response ratio. Food Addit. Contam. Part A.

[127] Wang B., Dong X.-S., Wang Z., Wang Y.-F., Hou Z.-Y. (2020). MEMS-Based Ionization Gas Sensors for VOCs with Array of Nanostructured Silicon Needles. ACS Sens..

[128] Liao W., Draper W.M., Perera S.K. (2008). Identification of Unknowns in Atmospheric Pressure Ionization Mass Spectrometry Using a Mass to Structure Search Engine. Anal. Chem..

[129] Osorio J., Dreolin N., Aznar M., Nerín C., Hancock P. (2019). Determination of volatile non intentionally added substances coming from a starch-based biopolymer intended for food contact by different gas chromatography-mass spectrometry approaches. J. Chromatogr. A.

[130] Pieke E.N., Granby K., Teste B., Smedsgaard J., Rivière G. (2018). Prioritization before risk assessment: The viability of uncertain data on food contact materials. Regul. Toxicol. Pharmacol..

[131] Severin I., Souton E., Dahbi L., Chagnon M.C. (2017). Use of bioassays to assess hazard of food contact material extracts: State of the art. Food Chem. Toxicol..

[132] Schilter B., Burnett K., Eskes C., Geurts L., Jacquet M., Kirchnawy C., Oldring P., Pieper G., Pinter E., Tacker M. (2019). Value and limitation of in vitro bioassays to support the application of the threshold of toxicological concern to prioritise unidentified chemicals in food contact materials. Food Addit. Contam. Part. A.

[133] Groh K.J., Geueke B., Muncke J. (2017). Food contact materials and gut health: Implications for toxicity assessment and relevance of high molecular weight migrants. Food Chem. Toxicol..

[134] Koster S., Rennen M., Leeman W., Houben G., Muilwijk B., van Acker F., Krul L. (2014). A novel safety assessment strategy for non-intentionally added substances (NIAS) in carton food contact materials. Food Addit. Contam. Part. A.

[135] Veyrand J., Marin-Kuan M., Bezencon C., Frank N., Guérin V., Koster S., Latado H., Mollergues J., Patin A., Piguet D. (2017). Integrating bioassays and analytical chemistry as an improved approach to support safety assessment of food contact materials. Food Addit. Contam. Part. A.

[136] Gelbke H.-P., Banton M., Block C., Dawkins G., Eisert R., Leibold E., Pemberton M., Puijk I.M., Sakoda A., Yasukawa A. (2019). Risk assessment for migration of styrene oligomers into food from polystyrene food containers. Food Chem. Toxicol..

[137] Eckardt M., Schneider J., Simat T.J. (2019). In vitro intestinal digestibility of cyclic aromatic polyester oligomers from polyethylene terephthalate (PET) and polybutylene terephthalate (PBT). Food Addit. Contam. Part A.

[138] Mertens B., Van Hoeck E., Blaude M.-N., Simon C., Onghena M., Vandermarken T., Van Langenhove K., Demaegdt H., Vandermeiren K., Covaci A. (2016). Evaluation of the potential health risks of substances migrating from polycarbonate replacement baby bottles. Food Chem. Toxicol..

[139] Koster S., Boobis A.R., Cubberley R., Hollnagel H.M., Richling E., Wildemann T., Würtzen G., Galli C.L. (2011). Application of the TTC concept to unknown substances found in analysis of foods. Food Chem. Toxicol..

[140] Kroes R., Renwick A.G., Cheeseman M., Kleiner J., Mangelsdorf I., Piersma A., Schilter B., Schlatter J., van Schothorst F., Vos J.G. (2004). Structure-based thresholds of toxicological concern (TTC): Guidance for application to substances present at low levels in the diet. Food Chem. Toxicol..

[141] Herrman J.L., Younes M. (1999). Background to the ADI/TDI/PTWI. Regul. Toxicol. Pharmacol..

[142] Tang X.Z., Kumar P., Alavi S., Sandeep K.P. (2012). Recent Advances in Biopolymers and Biopolymer-Based Nanocomposites for Food Packaging Materials. Crit. Rev. Food Sci. Nutr..

[143] Grujić R., Vujadinović D., Savanović D., Pellicer E., Nikolic D., Sort J., Baró M., Zivic F., Grujovic N., Grujic R., Pelemis S. (2017). Biopolymers as Food Packaging Materials. Advances in Applications of Industrial Biomaterials.

[144] Dintcheva N.T., Infurna G., Baiamonte M., D’Anna F. (2020). Natural Compounds as Sustainable Additives for Biopolymers. Polymers.

[145] Zimmermann L., Dombrowski A., Völker C., Wagner M. (2020). Are bioplastics and plant-based materials safer than conventional plastics? In vitro toxicity and chemical composition. Environ. Int..

[146] Polman E.M.N., Gruter G.-J.M., Parsons J.R., Tietema A. (2021). Comparison of the aerobic biodegradation of biopolymers and the corresponding bioplastics: A review. Sci. Total Environ..

[147] Simona J., Dani D., Petr S., Marcela N., Jakub T., Bohuslava T. (2021). Edible Films from Carrageenan/Orange Essential Oil/Trehalose—Structure, Optical Properties, and Antimicrobial Activity. Polymers.

[148] Wicochea-Rodríguez J.D., Chalier P., Ruiz T., Gastaldi E. (2019). Active Food Packaging Based on Biopolymers and Aroma Compounds: How to Design and Control the Release. Front. Chem..

[149] Asensio E., Montañés L., Nerín C. (2020). Migration of volatile compounds from natural biomaterials and their safety evaluation as food contact materials. Food Chem. Toxicol..

[150] Aznar M., Ubeda S., Dreolin N., Nerín C. (2019). Determination of non-volatile components of a biodegradable food packaging material based on polyester and polylactic acid (PLA) and its migration to food simulants. J. Chromatogr. A.

[151] Gavril G.-L., Wrona M., Bertella A., Świeca M., Râpă M., Salafranca J., Nerín C. (2019). Influence of medicinal and aromatic plants into risk assessment of a new bioactive packaging based on polylactic acid (PLA). Food Chem. Toxicol..

[152] Osorio J., Aznar M., Nerín C., Birse N., Elliott C., Chevallier O. (2020). Ambient mass spectrometry as a tool for a rapid and simultaneous determination of migrants coming from a bamboo-based biopolymer packaging. J. Hazard. Mater..

[153] Ubeda S., Aznar M., Alfaro P., Nerín C. (2019). Migration of oligomers from a food contact biopolymer based on polylactic acid (PLA) and polyester. Anal. Bioanal. Chem..

